# Serum P2X7 as a prognostic biomarker in acute supratentorial intracerebral hemorrhage: a two-center observational study

**DOI:** 10.3389/fneur.2025.1696189

**Published:** 2026-01-14

**Authors:** Xinle Chen, Hao Shan, Tiancheng Lu, Boren Zheng, Xiaoqiao Dong, Yulin Sun, Xiaojun Zhong, Guozheng Ying

**Affiliations:** 1 Department of Neurosurgery, Zhejiang Rongjun Hospital, Jiaxing, China; 2 The Fourth School of Clinical Medicine, Zhejiang Chinese Medical University, Hangzhou, China; 3 Department of Neurosurgery, Affiliated Hangzhou First People's Hospital, Westlake University School of Medicine, Hangzhou, China

**Keywords:** P2X7, intracerebral hemorrhage, prognosis, severity, biomarkers

## Abstract

**Introduction:**

P2X7 participates in neuroinflammation underlying acute brain injury. In this study, we investigated the prognostic role of serum P2X7 in acute intracerebral hemorrhage (ICH).

**Methods:**

This two-center observational analytic study enrolled 95 controls and 196 patients with acute supratentorial ICH. Serum P2X7 levels were measured at study entry in controls and at admission in all ICH patients, with serial measurements performed in a subgroup of 95 patients. Disease severity was assessed using the National Institutes of Health Stroke Scale (NIHSS) and hematoma volume. The primary neurological functional outcome was assessed using the modified Rankin Scale (mRS) at 6 months post-ICH. Multivariable analysis was performed to model the associations between P2X7 levels, disease severity, and final functional prognosis.

**Results:**

In patients with ICH, serum P2X7 levels were markedly elevated upon admission, peaked at day 3, and gradually declined through to day 10. Patient serum P2X7 levels remained notably higher than those of controls throughout the 10-day period. Serum P2X7 levels were linearly correlated with NIHSS scores, hematoma volume, mRS scores, and poor prognosis (mRS 3–6) under restricted cubic spline analysis. These associations persisted after adjustment for potential confounding factors. Sensitivity analyses, subgroup analyses, and receiver operating characteristic (ROC) curve analysis consistently demonstrated that serum P2X7 levels were robustly associated with poor prognosis. Applying calibration curve, decision curve, ROC curve, and model improvement rate analyses, a combined model integrating serum P2X7 levels, NIHSS scores, and hematoma volume demonstrated strong performance. Furthermore, serum P2X7 levels partially mediated the correlations of NIHSS scores and hematoma volume with poor prognosis.

**Discussion:**

Increased serum P2X7 levels after ICH are markedly associated with disease severity and poor prognosis. Moreover, P2X7 may partially mediate the relationship between ICH severity and prognosis, underscoring its potential as a prognostic biomarker.

## 1 Introduction

Spontaneous intracerebral hemorrhage (ICH) is a common neurological disease that primarily affects the elderly (1). Its incidence gradually increases with population aging (2). The main pathological bases are hypertensive arteriolosclerosis (leading to deep hemorrhages) and cerebral amyloid angiopathy (leading to lobar hemorrhages) (3). Hematoma can directly compress neurons and glia, while concurrent bleeding triggers secondary brain injury characterized by inflammatory responses, oxidative stress, and neuronal apoptosis, which together cause disruption of the blood–brain barrier. This breakdown aggravates cerebral edema, damages neurons, impairs neurological function, and may ultimately cause patient death (4–6). Severity assessment is a critical step in the clinical management of ICH, as disease severity is intimately associated with patient outcome (7, 8). Conventionally, the National Institutes of Health Stroke Scale (NIHSS) and hematoma volume are used to evaluate ICH severity (9–11), while the modified Rankin Scale (mRS) is applied to assess patients’ neurological functional status (12, 13). Because blood samples are easily accessible, biomarkers such as Apo-J, vascular endothelial growth factor, glial fibrillary acidic protein, and S100 calcium binding protein B (S100B) have received considerable attention for their prognostic significance (14–16).

Purinergic signaling is implicated in innate and adaptive immunity (17–19). P2X7 is a member of the P2X purinoreceptor family, activated by high concentrations of extracellular ATP (20–22). In the central nervous system, P2X7 is extensively expressed on the surface of several cell types, including astrocytes, neurons, and microglia (23–25). P2X7 is a prominent neuroinflammatory receptor in acute brain injury, with expression levels markedly upregulated in brain tissue (26–28). In response to acute ischemic, hemorrhagic, or traumatic brain injury, P2X7 acts as a major driver of neuroinflammation, disrupting the blood–brain barrier, aggravating brain edema, and inducing neuronal apoptosis (29–32). Inhibition of P2X7 attenuates these pathological effects (33–42). Thus, P2X7 has emerged as a potential therapeutic target in brain diseases (43–47). Also, P2X7 may serve as a biomarker of acute brain injury. In this study, we aimed to investigate the temporal kinetics of serum P2X7 levels and to evaluate its prognostic significance in ICH.

## 2 Materials and methods

### 2.1 Study design

**Figure 1:**
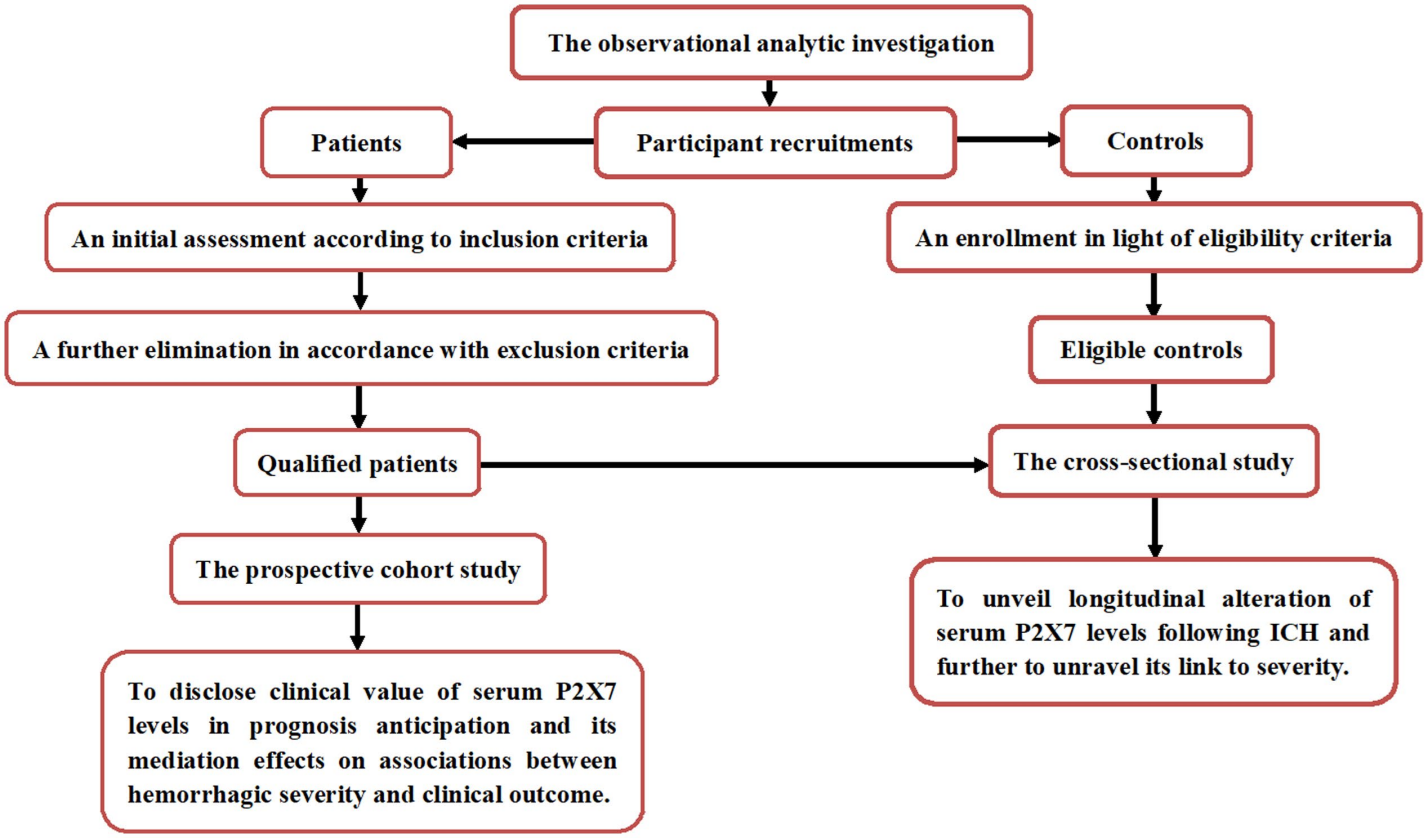
Flow chart of study design and participant enrollment. The study included a prospective cohort and a cross-sectional sub-study, aiming to assess longitudinal changes in serum P2X7 levels after ICH and its prognostic value. ICH, intracerebral hemorrhage.

This two-center observational analytical study was conducted between August 2019 and September 2023 at the Zhejiang Rongjun Hospital (Jiaxing, China) and Affiliated Hangzhou First People’s Hospital, Westlake University School of Medicine (Hangzhou, China). The study was divided into two segments, a cross-sectional sub-study and a prospective cohort sub-study (Figure 1). Based on the eligibility criteria, controls and patients with ICH were recruited. Inclusion criteria were: (1) radiological confirmation of supratentorial intraparenchymal bleeding; (2) age ≥18 years; (3) transfer of patients to hospital within 24 h post-stroke; (4) first-episode stroke; (5) hemorrhage not attributable to secondary factors; (6) non-surgical intervention of hematoma; and (7) preadmission mRS score equal to 0. Exclusion criteria included (1) presence of other neuropsychiatric conditions, including ischemic or hemorrhagic stroke, intracranial mass lesions, moderate to severe brain trauma, central nervous system infections, schizophrenia, multiple sclerosis, or other significant neurological or psychiatric disorders; or (2) presence of other severe illnesses or specific circumstances, including malignancies, systemic disorders, other organ failure, use of immunosuppressive agents, infection within the past month, requirement for mechanical ventilation, loss to follow-up, inadequate clinical recordings, refusal to participate, or unsuitable blood specimens. Eligibility criteria for control selection included (1) the absence of chronic diseases, including hypertension, diabetes mellitus, hyperuricemia, and related conditions; and (2) normal results on routine clinical tests, such as blood leukocyte counts, blood glucose levels, and chest radiography. Finally, a total of 196 patients and 95 controls were enrolled. Admission blood samples were obtained from all 196 patients, of whom 95 voluntarily contributed additional serial blood specimens. Blood samples from all 95 controls were collected at study entry. Sample size calculations showed that 108 patients were sufficient for prognostic analysis and 78 patients were adequate for evaluating changes in serum P2X7 levels. Thus, the number of patients enrolled was adequate to permit analysis of temporal trends in serum P2X7 levels after ICH and its prognostic implications.

### 2.2 Ethical considerations

This study conformed to the Declaration of Helsinki and its later amends. The study protocol was approved by the Ethics Committees of the Zhejiang Rongjun Hospital (approval number, RJY076) and Affiliated Hangzhou First People’s Hospital, Westlake University School of Medicine (approval number, SYY058-01). Patients’ legal proxies and controls themselves were notified of the study details and independently signed informed consent forms.

### 2.3 Data collection and outcome assessment

Demographical data (age and gender), lifestyle habits (cigarette smoking and alcohol consumption), chronic diseases (hypertension, diabetes mellitus, dyslipidemia, chronic obstructive pulmonary disease, ischemic heart disease, and hyperuricemia), and use of specific drugs (statins, anticoagulants, and antiplatelet agents) were recorded. Using a non-invasive technique, systolic arterial blood pressure and diastolic arterial blood pressure were measured at patient admission. Neurological deficits were evaluated using the NIHSS (11). According to initial head computed tomography images, hematoma size was calculated using the equation: 0.5 × a × b × c (48). Intraventricular or subarachnoidal extension of hemorrhage was registered. Hematoma sites were of two types: superficial or deep. Neurological status was appraised using the modified Rankin Scale (mRS). mRS ranges from 0 to 6, with higher scores indicating worse status, and a score of 6 denoting death (12, 13). Via telephone visits, neurological deficits at 6 months post-ICH were assessed using the mRS through structured interviews, and scores from 3 to 6 were defined as poor prognosis (12, 13).

### 2.4 Blood sampling and processing and biomarker measurements

Blood samples were collected from all patients upon admission, on days 1, 3, 5, 7 and 10 following ICH from a subset of patients and at recruitment from all controls. Blood was drawn via the antecubital vein into separation gel-containing tubes (5 mL; Shengshi Dongtang Jiangsu Biotechnology Co., Ltd.). The specimens were centrifuged at 2,000 × g for 10 min to remove cells and debris. The supernatants were extracted, rapidly transferred to microcentrifuge tubes, and preserved at −80 °C until use. All samples were thawed within 3 months of collection for measurement of serum P2X7 levels using an enzyme-linked immunosorbent assay kit (CUSABIO, Wuhan, China, Cat. No. CSB-EL017325HU). The assay’s detection range was 25 to1,600 pg/mL, with intra-assay coefficients of variation <8%, interassay coefficients of variation <10%, and sensitivity of 6.25 pg/mL. Measurements were performed in duplicate according to the manufacturer’s instructions by a trained technician who was blinded to clinical information. The two measurements were averaged for subsequent statistical analyses.

### 2.5 Statistical analysis

Statistical analyses were performed using SPSS 23.0 (SPSS Inc., Chicago, IL, United States). Variables were presented as counts (percentages), means (standard deviations), or medians (25th–75th percentiles) as appropriate. For group comparisons, the Chi-square test, Fisher’s exact test, independent-samples Student’s *t*-test, Mann–Whitney *U* test, Kruskal–Wallis test, or one-way analysis of variance (ANOVA) was applied, as deemed suitable. Bivariate correlations were assessed using Spearman’s rank correlation test. Serum P2X7 levels, mRS scores, and poor prognosis were selected as dependent variables. Multivariate methods included binary logistic regression, multivariate linear regression, and ordinal regression analyses. Factors that were significant on univariate analyses were entered into multivariate models to screen independently associated variables. R software v3.5.1^1^ was used for model establishment and validation. The combined model integrating independent predictors of poor prognosis was illustrated using a nomogram, verified for goodness of fit with calibration curve analysis, and assessed for clinical validity through decision curve analysis. E-values were computed for sensitivity analyses (49). Restricted cubic spline (RCS) analysis was applied to flexibly model the potentially non-linear relationship between the variables (50).

Subgroup analyses were performed to assess interaction effects (51). Variance inflation factors (VIF) were calculated for judging multicollinearity in regression models, with VIF < 10 indicating absence of multicollinearity (52). Model improvement rate was reflected by net reclassification improvement and integrated discrimination improvement (53). Mediation analysis was carried out to determine whether serum P2X7 levels mediated the association between bleeding severity and clinical outcome (54). MedCalc v20 (MedCalc Software, Ltd., Ostend, Belgium) was used to plot receiver operating characteristic (ROC) curves to evaluate discrimination effectiveness. For sample size estimation in comparing serum P2X7 levels, the type 1 error (*α*) was set at 0.05, test power (1-*β*) at 0.95, and effect size at 0.8. The accuracy of sample size estimation was subsequently assessed using *a priori* power analysis in G*Power v3.1.9.4 (Heinrich-Heine Universität Düsseldorf, Germany). Because this was a prospective cohort study, missing data were rare; only three patients had incomplete clinical data and were excluded from the study. Dual measurements of serum P2X7 levels were assessed for concordance using Bland–Altman plots and the intraclass correlation coefficient. In all analyses, differences were considered statistically significant when two-sided *p*-values were less than 0.05.

## 3 Results

### 3.1 Participant features

A total of 261 patients were initially evaluated according to the pre-established eligibility criteria. Of these, 65 were excluded: 24 due to neuropsychiatric disorders, 32 due to severe comorbid organ diseases, 1 due to loss to follow-up, 3 due to incomplete clinical records, 1 due to refusal to participate, and 4 due to inadequate blood specimens. Ultimately, 196 patients were eligible for inclusion in the study. All 196 patients provided blood samples at admission and 95 of them voluntarily contributed subsequent serial samples. Concurrently, 95 individuals were recruited as controls. As listed in Table 1, age, gender, cigarette smoking, and alcohol drinking did not differ significantly among controls, all patients, and those who underwent serial measurements. All other baseline parameters were also not significantly different between all patients and those with serial measurements (*p* > 0.05).

**Table 1:** Baseline characteristics of controls and patients with acute ICH.

Variable	All patients	Patients with serial blood sampling	Controls	*p*
Sample size	196	95	95	
Age (years)	63.6 ± 10.5	64.1 ± 10.6	61.4 ± 10.9	0.167
Gender (male/female)	113/83	52/43	53/42	0.884
Cigarette smoking	66 (33.7%)	27 (28.4%)	30 (31.6%)	0.664
Alcohol drinking	68 (34.7%)	30 (31.6%)	27 (28.4%)	0.552
Hypertension	127 (64.8%)	65 (68.4%)		0.540
Diabetes mellitus	34 (17.3%)	18 (18.9%)		0.738
Dyslipidemia	62 (31.6%)	30 (31.6%)		0.993
COPD	8 (4.1%)	7 (7.4%)		0.234
Ischemic heart disease	13 (6.6%)	9 (9.5%)		0.390
Hyperuricemia	23 (11.7%)	13 (13.7%)		0.636
Previous statin use	45 (23.0%)	23 (24.2%)		0.813
Previous anticoagulant use	13 (6.6%)	5 (5.3%)		0.649
Previous antiplatelet use	23 (11.7%)	11 (11.6%)		0.969
Admission time (h)	9.0 (5.0–14.3)	7.2 (3.9–14.0)		0.135
Sampling time (h)	9.8 (6.0–15.5)	8.3 (4.5–15.0)		0.143
Systolic arterial pressure (mmHg)	140.8 ± 28.8	141.3 ± 29.9		0.883
Diastolic arterial pressure (mmHg)	88.2 ± 12.9	88.6 ± 13.2		0.820
Hematoma location (superficial/deep)	55/141	28/67		0.802
Intraventricular hematoma	28 (14.3%)	13 (13.7%)		0.890
Subarachnoidal hematoma	8 (4.1%)	4 (4.2%)		0.959
NIHSS score	8 (5–12)	9 (5–13)		0.496
Hematoma volume (mL)	11 (9–20)	13 (9–21)		0.563
Blood leucocyte count (×10^9^/L)	6.1 (4.2–8.9)	6.3 (4.2–9.6)		0.867
Blood glucose level (mmol/L)	9.1 (7.3–11.5)	9.4 (7.4–11.9)		0.410
Six-month mRS (continuous form)	2 (1–4)	2 (2–4)		0.702
Six-month mRS (categorical form)				0.993
0	16	8		
1	36	15		
2	56	28		
3	30	13		
4	22	13		
5	25	12		
6	11	6		
Six-month poor prognosis (n)	88 (44.9%)	44 (46.3%)		0.820

As appropriate, variables are reported as frequency (proportion), mean ± standard deviation, or median (25th–75th percentiles). Statistical approaches included chi-square test, Fisher exact test, Student’s 𝑡-test, Mann–Whitney test, and one-way ANOVA. COPD, chronic obstructive pulmonary disease; NIHSS: National Institutes of Health Stroke Scale; mRS, modified Rankin Scale.

### 3.2 Temporal kinetics of serum P2X7 levels and correlation with ICH severity

**Figure 2:**
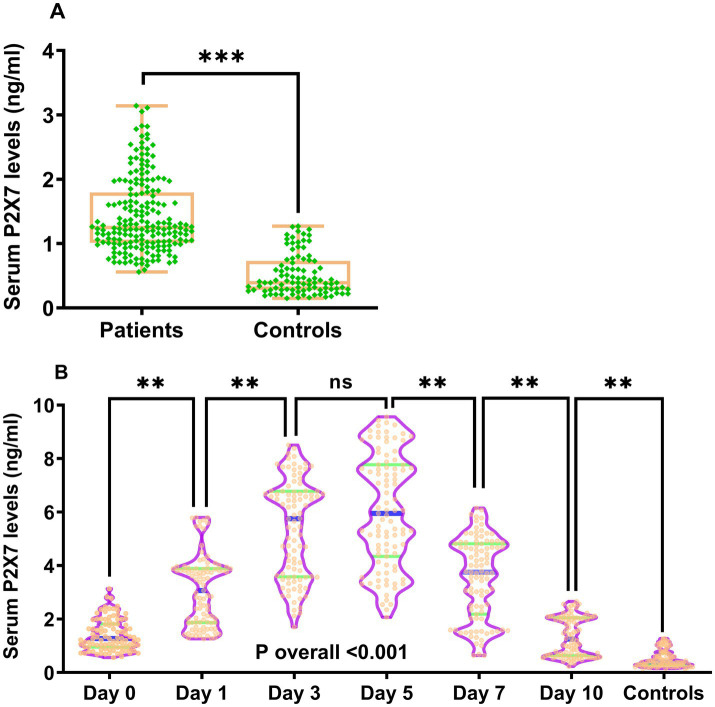
Temporal kinetics of serum P2X7 levels following acute ICH. **(A)** At admission, patient serum P2X7 levels were significantly higher than those of controls (*p* < 0.001). **(B)** In patients with continuous samplings, serum P2X7 levels rose promptly on arrival, peaked at day 3, remained stable at day 5, and gradually declined thereafter, yet stayed markedly higher than controls within 10 days (*p* for trend < 0.001). ns, Non-significant; ***p < 0.001; **p < 0.01.

Double measurements of serum P2X7 levels showed satisfactory concordance in all detections (intraclass correlation coefficient = 0.993; Supplementary Figure 1). At admission, serum P2X7 levels were substantially higher in patients than in controls (*p* < 0.001; Figure 2A). In patients undergoing serial sampling, serum P2X7 levels were markedly elevated at admission, continued to rise on day 1, reached a peak on day 3, plateaued at day 5, and gradually declined from day 7 to day 10. Overall, serum P2X7 levels remained significantly higher in patients than in controls throughout the 10-day period (*p* < 0.001; Figure 2B). Baseline NIHSS scores and hematoma volumes in patients undergoing serial sampling were strongly correlated with serum P2X7 levels at admission and on days 1, 3, 5, 7, and 10 after acute ICH, with correlation coefficients ranging from 0.44 to 0.62 (all *p* < 0.001; Supplementary Figure 2). In all patients, admission serum P2X7 levels were linearly associated with initial NIHSS scores (*p* for nonlinearity >0.05; Supplementary Figure 3A) and hematoma volume (*p* for nonlinearity >0.05; Supplementary Figure 3B) using the RCS method. Admission serum P2X7 levels were also highly correlated to initial NIHSS scores (*p* < 0.001; Supplementary Figure 4A) and hematoma volume (*p* < 0.001; Supplementary Figure 4B) based on Spearman’s rank correlation analysis. Furthermore, admission serum P2X7 levels in all patients were significantly associated with age, diabetes, and intraventricular extension of bleeding (all *p* < 0.05; Table 2). When these five factors were included in the multivariate model, NIHSS scores (*β* = 0.042; 95% confidence interval (CI) = 0.019–0.064; VIF = 2.752; *p* = 0.001) and hematoma volume (*β* = 0.024; 95% CI = 0.012–0.037; VIF = 2.807; *p* = 0.001) remained independently associated with admission serum P2X7 levels in patients with ICH.

**Table 2:** Bivariate correlations between serum P2X7 levels and clinical variables in patients with acute ICH.

Variable	Spearman test	
	*ρ*	*p*
Age (years)	0.145	0.042
Gender (male/female)	0.011	0.873
Cigarette smoking	0.120	0.093
Alcohol drinking	0.095	0.185
Hypertension	−0.018	0.800
Diabetes mellitus	0.171	0.017
Dyslipidemia	0.001	0.995
COPD	0.020	0.780
Ischemic heart disease	−0.044	0.573
Hyperuricemia	−0.033	0.647
Previous statin use	0.069	0.338
Previous anticoagulant use	−0.040	0.567
Previous antiplatelet use	0.048	0.501
Admission time (h)	0.092	0.202
Sampling time (h)	0.101	0.158
Systolic arterial pressure (mmHg)	−0.006	0.937
Diastolic arterial pressure (mmHg)	−0.014	0.848
Hematoma location (superficial/deep)	−0.010	0.889
Intraventricular hematoma	0.246	0.001
Subarachnoidal hematoma	−0.018	0.797
NIHSS score	0.595	<0.001
Hematoma volume (ml)	0.627	<0.001
Blood leucocyte count (×10^9^/L)	0.045	0.533
Blood glucose level (mmol/L)	0.140	0.051

COPD, chronic obstructive pulmonary disease; NIHSS: National Institutes of Health Stroke Scale.

### 3.3 Serum P2X7 levels and neurological functional status at 6 months post-ICH

The mRS scores of patients undergoing serial sampling were strongly correlated with serum P2X7 levels at all time points after ICH, with correlation coefficients ranging from 0.53 to 0.66 (all *p* < 0.001; Supplementary Figure 2). In all 196 patients, admission serum P2X7 levels and mRS scores were linearly associated on RCS analysis (*p* for nonlinearity >0.05; Supplementary Figure 3C) and strongly correlated on Spearman’s rank analysis (*p* < 0.001; Supplementary Figure 4C). In addition, in all ICH patients, age, diabetes, intraventricular expansion of hematoma, NIHSS score, hematoma volume, blood leucocyte counts, and blood glucose levels were all highly correlated with mRS scores (all *p* < 0.05; Table 3). When all significantly correlated parameters were included in the multivariate model, admission serum P2X7 levels (*β* = 1.072; 95% CI = 0.658–1.486; VIF = 2.005; *p* = 0.008), NIHSS scores (*β* = 0.107; 95% CI = 0.041–0.172; VIF = 2.967; *p* = 0.001), and hematoma volume (*β* = 0.044; 95% CI = 0.015–0.073; VIF = 1.883; *p* = 0.003) remained independently associated with mRS scores.

**Table 3:** Bivariate correlations between 6-month mRS scores and clinical factors in patients with acute ICH.

Variable	Spearman test	
	*ρ*	*p*
Age (years)	0.227	0.001
Gender (male/female)	−0.114	0.111
Cigarette smoking	−0.038	0.601
Alcohol drinking	−0.048	0.501
Hypertension	−0.047	0.516
Diabetes mellitus	0.167	0.019
Dyslipidemia	0.012	0.869
COPD	0.120	0.094
Ischemic heart disease	0.008	0.914
Hyperuricemia	0.069	0.337
Previous statin use	0.085	0.235
Previous anticoagulant use	−0.024	0.735
Previous antiplatelet use	0.059	0.409
Admission time (h)	−0.055	0.443
Sampling time (h)	−0.042	0.557
Systolic arterial pressure (mmHg)	0.018	0.799
Diastolic arterial pressure (mmHg)	−0.015	0.838
Hematoma site (superficial/deep)	−0.051	0.480
Intraventricular hematoma	0.324	<0.001
Subarachnoidal hematoma	0.061	0.399
NIHSS score	0.616	<0.001
Hematoma volume (mL)	0.632	<0.001
Blood leucocyte count (×10^9^/L)	0.160	0.025
Blood glucose level (mmol/L)	0.195	0.006
Serum P2X7 level (ng/mL)	0.592	<0.001

COPD, chronic obstructive pulmonary disease; NIHSS: National Institutes of Health Stroke Scale.

Patients with serial blood sampling were allocated to seven subgroups based on mRS scores. At all sampling time points, serum P2X7 levels differed significantly among subgroups, with higher scores corresponding to higher P2X7 levels (all *p* < 0.001; Supplementary Figure 5). At admission, serum P2X7 levels in all patients increased progressively with higher mRS scores (0–6); (*p* for trend < 0.001; Supplementary Figure 6). As listed in Table 4, age, diabetes, blood accumulation within the intraventricular space, NIHSS scores, hematoma volume, blood glucose levels, and admission P2X7 levels were significantly different among the seven subgroups (all *p* < 0.05). When the preceding seven variables were integrated into an ordinal regression model, admission serum P2X7 levels (odds ratio (OR) = 4.150; 95% CI = 2.184–7.885; VIF = 2.821; *p* = 0.009), NIHSS scores (OR = 1.196; 95% CI = 1.082–1.323; VIF = 2.129; *p* = 0.001), and hematoma volume (OR = 1.081; 95% CI = 1.036–1.129; VIF = 2.347; *p* = 0.002) remained independently associated with ordinal mRS scores.

**Table 4:** Clinical factors across seven subgroups defined by 6-month modified mRS scores.

Variable	mRS score							*p*
	0	1	2	3	4	5	6	
Age (years)	59 (51–64)	60 (53–72)	64 (55–70)	62 (52–72)	63 (60–72)	71 (55–78)	72 (64–80)	0.016
Gender (male/female)	10/6	22/14	36/20	17/13	11/11	12/13	5/6	0.743
Cigarette smoking	6 (37.5%)	13 (36.1%)	20 (35.7%)	9 (30.0%)	5 (22.7%)	8 (32.0%)	5 (45.5%)	0.881
Alcohol drinking	7 (43.8%)	13 (36.1%)	20 (35.7%)	9 (30.0%)	6 (27.3%)	9 (36.0%)	4 (36.4%)	0.960
Hypertension	9 (56.3%)	25 (69.4%)	41 (73.2%)	17 (56.7%)	10 (45.5%)	19 (76.0%)	6 (54.5%)	0.176
Diabetes mellitus	2 (12.5%)	6 (16.7%)	4 (7.1%)	4 (13.3%)	8 (36.4%)	6 (24.0%)	4 (36.4%)	0.041
Dyslipidemia	4 (25.0%)	10 (27.8%)	20 (35.7%)	11 (36.7%)	9 (40.9%)	3 (12.0%)	5 (45.5%)	0.264
COPD	1 (6.3%)	1 (2.8%)	0 (0.0%)	0 (0.0%)	3 (13.6%)	1 (4.0%)	2 (18.2%)	0.053
Ischemic heart disease	1 (6.3%)	2 (5.6%)	4 (7.1%)	3 (10.0%)	1 (4.5%)	0 (0.0%)	2 (18.2%)	0.551
Hyperuricemia	1 (6.3%)	3 (8.3%)	6 (10.7%)	6 (20.0%)	4 (18.2%)	0 (0.0%)	3 (27.3%)	0.139
Previous statin use	0 (0.0%)	7 (19.4%)	15 (26.8%)	10 (33.3%)	7 (31.8%)	3 (12.0%)	3 (27.3%)	0.121
Previous anticoagulant use	2 (12.5%)	1 (2.8%)	5 (8.9%)	2 (6.7%)	1 (4.5%)	1 (4.0%)	1 (9.1%)	0.837
Previous antiplatelet use	1 (6.3%)	1 (2.8%)	11 (19.6%)	4 (13.3%)	1 (4.5%)	3 (12.0%)	2 (18.2%)	0.220
Admission time (h)	7.1 (4.6–14.9)	11.8 (6.0–16.4)	9.0 (4.8–14.6)	8.6 (5.9–13.5)	5.8 (3.0–8.0)	8.0 (5.5–11.5)	11.7 (9.1–15.6)	0.178
Sampling time (h)	7.7 (5.8–15.4)	12.3 (7.3–17.3)	10.0 (6.0–15.5)	9.5 (6.5–16.0)	6.9 (4.0–9.0)	9.0 (6.3–13.0)	13.0 (9.8–16.2)	0.204
Systolic arterial pressure (mmHg)	131 (121–171)	127 (117–139)	136 (119–181)	128 (118–138)	133 (126–191)	133 (115–175)	130 (119–150)	0.225
Diastolic arterial pressure (mmHg)	88 (77–105)	85 (78–97)	90 (83–101)	83 (76–89)	87 (83–102)	85 (76–105)	85 (83–94)	0.263
Hematoma site (superficial/deep)	5/11	11/25	18/38	5/25	6/16	7/18	3/8	0.858
Intraventricular hematoma	0 (0.0%)	1 (2.8%)	4 (7.1%)	6 (20.0%)	5 (22.7%)	8 (32.0%)	4 (36.4%)	0.001
Subarachnoidal hematoma	0 (0.0%)	1 (2.8%)	3 (5.4%)	1 (3.3%)	0 (0.0%)	3 (12.0%)	0 (0.0%)	0.367
NIHSS score	4 (1–6)	5 (3–8)	8 (6–11)	9 (6–13)	11 (8–13)	14 (12–16)	13 (11–14)	<0.001
Hematoma volume (mL)	6 (4–8)	8 (5–10)	11 (9–19)	13 (9–20)	18 (13–22)	24 (16–30)	19 (16–20)	<0.001
Blood leucocyte count (×10^9^/L)	6.4 (4.6–7.2)	5.4 (4.4–6.4)	5.5 (4.2–9.3)	5.8 (3.6–9.6)	7.3 (5.9–9.6)	6.5 (4.0–8.9)	9.6 (7.6–11.6)	0.125
Blood glucose level (mmol/L)	8.3 (6.6–9.5)	8.2 (5.7–10.0)	8.8 (7.3–10.4)	11.0 (7.5–13.9)	9.2 (7.5–13.7)	10.2 (8.6–15.0)	7.9 (3.4–10.9)	0.004
Serum P2X7 level (ng/mL)	0.7 (0.7–0.8)	1.2 (1.0–1.3)	1.2 (1.0–1.4)	1.1 (1.0–2.0)	1.5 (1.1–1.8)	2.1 (1.8–2.5)	2.1 (1.8–2.4)	<0.001

Variables are presented as count (percentage) or median (interquartile range, 25th–75th percentiles). Statistical analyses were performed using chi-square and Kruskal−Wallis *H* tests. COPD, chronic obstructive pulmonary disease; NIHSS, National Institutes of Health Stroke Scale.

**Figure 3:**
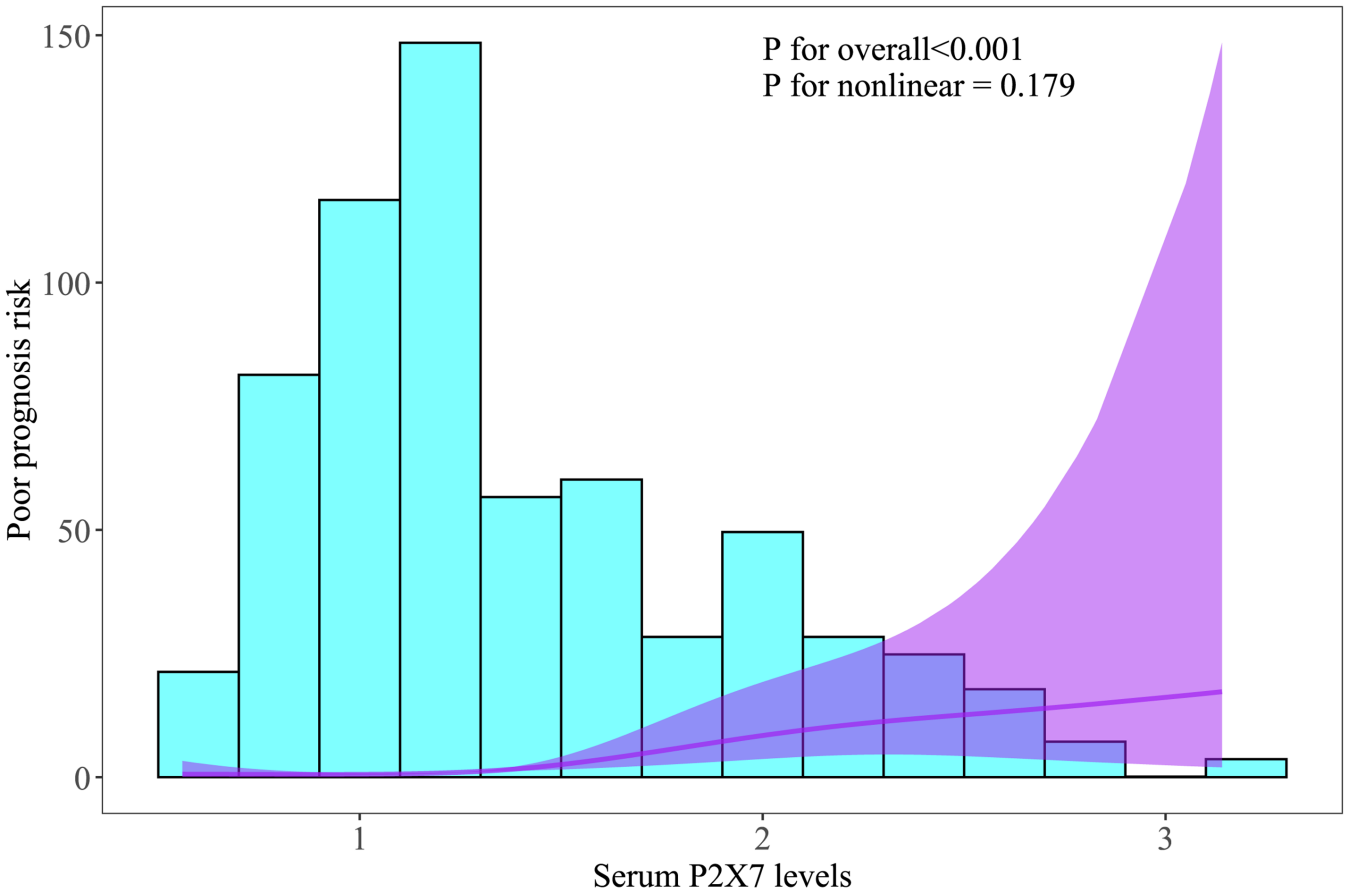
Linear relationship between admission serum P2X7 levels and probability of poor prognosis after acute ICH. Restricted cubic spline analysis showed a dose-effect correlation between admission P2X7 levels and 6-month poor prognosis risk (*p* > 0.05).

**Figure 4:**
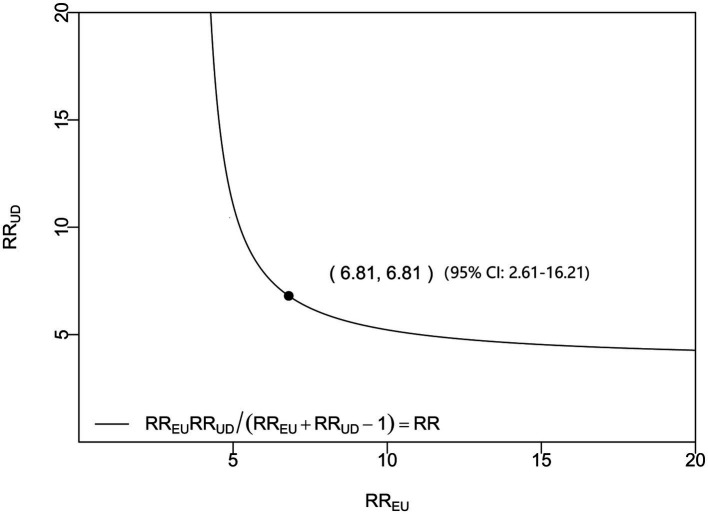
Sensitivity analysis of admission serum P2X7 levels and poor prognosis in acute ICH. Based on the odds ratio (OR), the *E*-value was 6.81, indicating a robust prognostic association.

In patients with serial samplings, serum P2X7 levels were at all time points markedly higher in cases presenting with poor prognosis than in those having good prognosis (all *p* < 0.001; Supplementary Figure 7). Moreover, the prognostic predictive ability of admission serum P2X7 levels was comparable to that at the other time points, as shown by ROC curve analysis (all *p* > 0.05; Supplementary Figure 8). Among all ICH patients, those with poor prognosis showed significantly elevated admission serum P2X7 levels compared with the remaining cases (*p* < 0.001; Supplementary Figure 9A). ROC curve analysis showed that admission serum P2X7 levels effectively predicted poor prognosis, and the optimal cutoff value was determined using the Youden index (Supplementary Figure 9B). Applying RCS analysis, admission serum P2X7 showed a linear correlation with probability of poor prognosis (*p* for nonlinearity > 0.05; Figure 3). As listed in Table 5, compared with patients with good prognosis, those with poor prognosis were significantly older, more likely to have diabetes and intraventricular hematoma, and had higher NIHSS scores, hematoma volumes, blood glucose levels, and admission serum P2X7 levels (all *p* < 0.05). When the abovementioned variables were inputted into a binary logistic regression model, admission serum P2X7 levels (OR = 3.237; 95% CI = 1.346–7.778; VIF = 2.821; *p* = 0.010), NIHSS score (OR = 1.173; 95% CI = 1.025–1.343; VIF = 3.773; *p* = 0.004), and hematoma volume (OR = 1.079; 95% CI = 1.020–1.142; VIF = 3.009; *p* = 0.008) remained independently predictive of poor prognosis. The overall model demonstrated good calibration (Hosmer–Lemeshow test, *p* = 0.481) and acceptable accuracy (Brier score, 0.219) (see Figure 4).

**Table 5:** Factors in connection with poor prognosis 6 months after acute ICH.

Variable	Poor prognosis	Good prognosis	*p*
Age (years)	65.2 ± 10.7	62.2 ± 10.2	0.047
Gender (male/female)	45/43	68/40	0.096
Cigarette smoking	27 (30.7%)	39 (36.1%)	0.424
Alcohol drinking	28 (31.8%)	40 (37.0%)	0.445
Hypertension	52 (59.1%)	75 (69.4%)	0.131
Diabetes mellitus	22 (25.0%)	12 (11.1%)	0.011
Dyslipidemia	28 (31.8%)	34 (31.5%)	0.960
COPD	6 (6.8%)	2 (1.9%)	0.080
Ischemic heart disease	6 (6.8%)	7 (6.5%)	0.925
Hyperuricemia	13 (14.8%)	10 (9.3%)	0.233
Previous statin use	23 (26.1%)	22 (20.4%)	0.340
Previous anticoagulant use	5 (5.7%)	8 (7.4%)	0.629
Previous antiplatelet use	10 (11.4%)	13 (12.0%)	0.884
Admission time (h)	8.0 (4.6–12.8)	9.4 (5.0–15.6)	0.282
Sampling time (h)	9.0 (5.5–14.2)	10.3 (6.2–16.3)	0.350
Systolic arterial pressure (mmHg)	138.9 ± 27.7	142.3 ± 29.7	0.423
Diastolic arterial pressure (mmHg)	87.2 ± 12.1	89.1 ± 13.5	0.329
Hematoma site (superficial/deep)	21/67	34/74	0.238
Intraventricular hematoma	23 (26.1%)	5 (4.6%)	<0.001
Subarachnoidal hematoma	4 (4.5%)	4 (3.7%)	0.767
NIHSS score	12 (8–14)	6 (4–9)	<0.001
Hematoma volume (mL)	19 (12–23)	9 (6–13)	<0.001
Blood leucocyte count (×10^9^/L)	7.1 (4.0–9.6)	5.5 (4.3–7.3)	0.062
Blood glucose level (mmol/L)	9.5 (7.7–13.9)	8.5 (6.6–10.1)	0.002
Serum P2X7 level (ng/mL)	1.8 (1.1–2.2)	1.2 (0.9–1.3)	<0.001

Variables are presented as count (percentage), mean ± standard deviation, or median (interquartile range, 25^th^–75^th^ percentiles). The chi-square test, Fisher’s exact test, Student’s 𝑡-test, or Mann–Whitney test were used for data comparison. COPD, chronic obstructive pulmonary disease; NIHSS, National Institutes of Health Stroke Scale.

**Figure 5:**
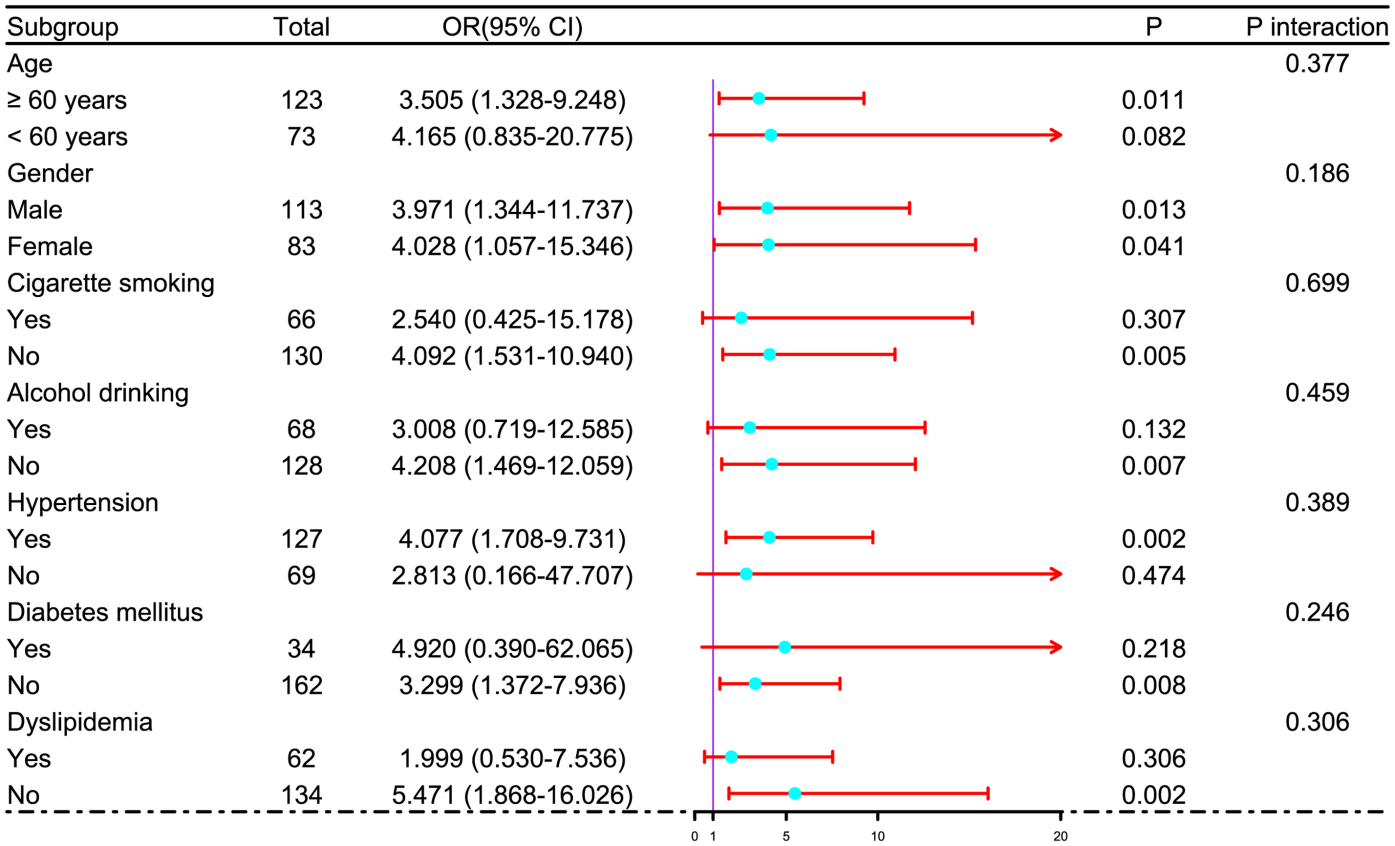
Subgroup analysis of admission serum P2X7 levels and poor prognosis after acute ICH. No significant interactions were observed between admission P2X7 and conventional variables (age, gender, cigarette smoking, etc.) in relation to poor prognosis (all *p* for interaction > 0.05).

**Figure 6:**
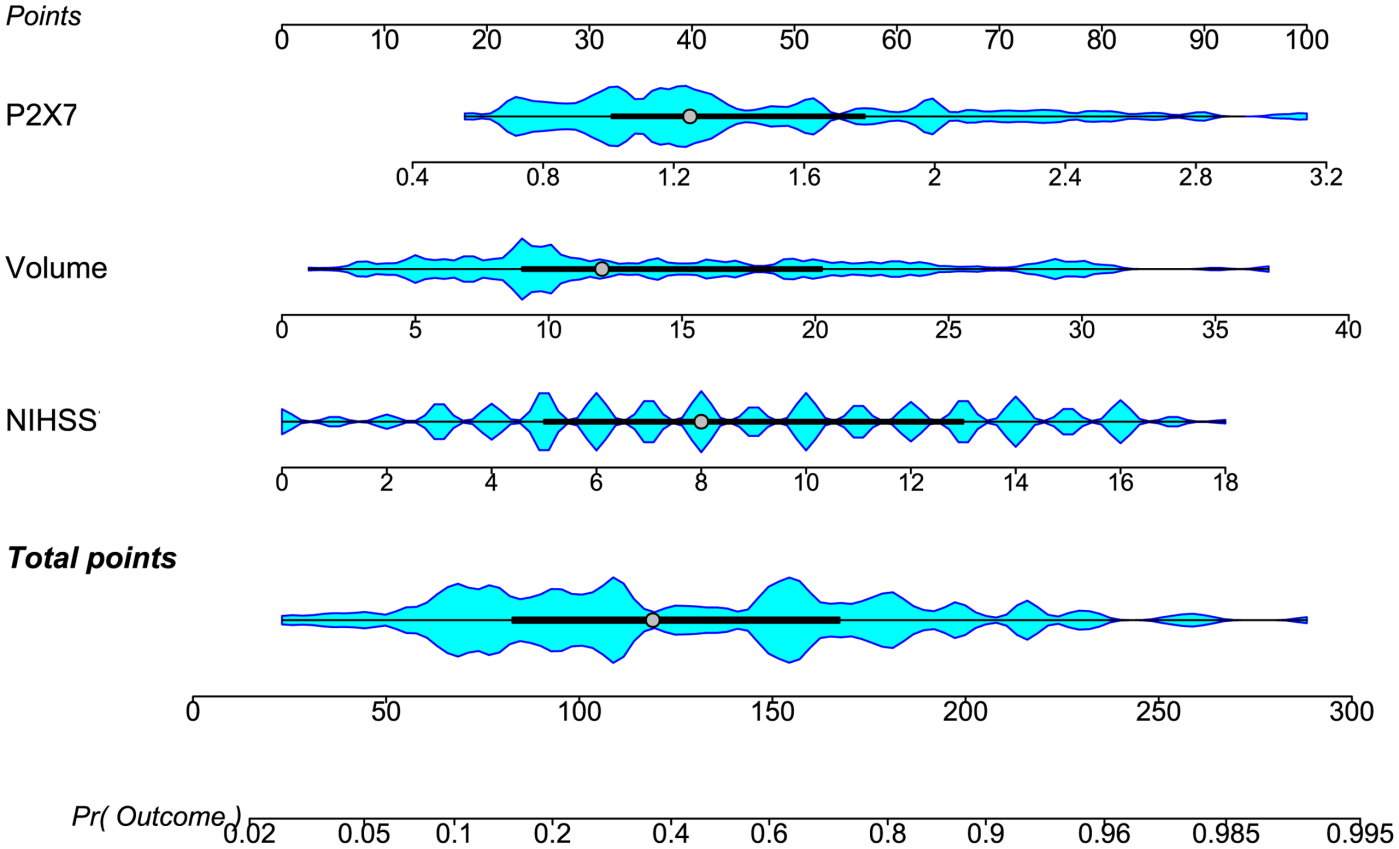
Nomogram of the prognostic model for acute ICH. Three independent predictors, NIHSS score, admission serum P2X7, and hematoma volume, were combined to construct the model. The nomogram assigns each predictor a risk score; summed scores yield a total score corresponding to risk probability. NIHSS: National Institutes of Health Stroke Scale; volume, hematoma volume.

**Figure 7:**
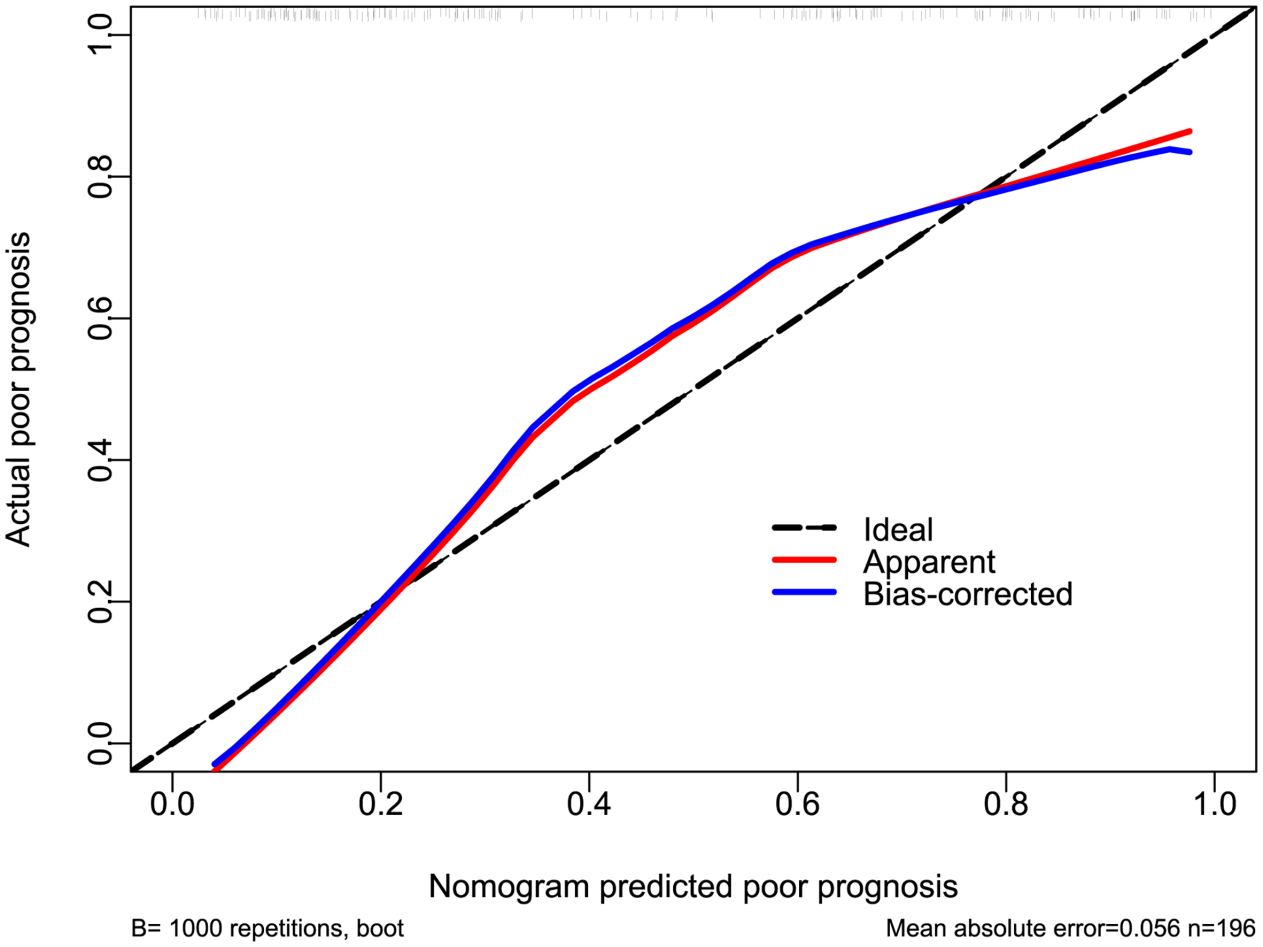
Calibration curve of the prognostic model in acute ICH. The nomogram showed satisfactory goodness of fit for predicting poor prognosis.

**Figure 8:**
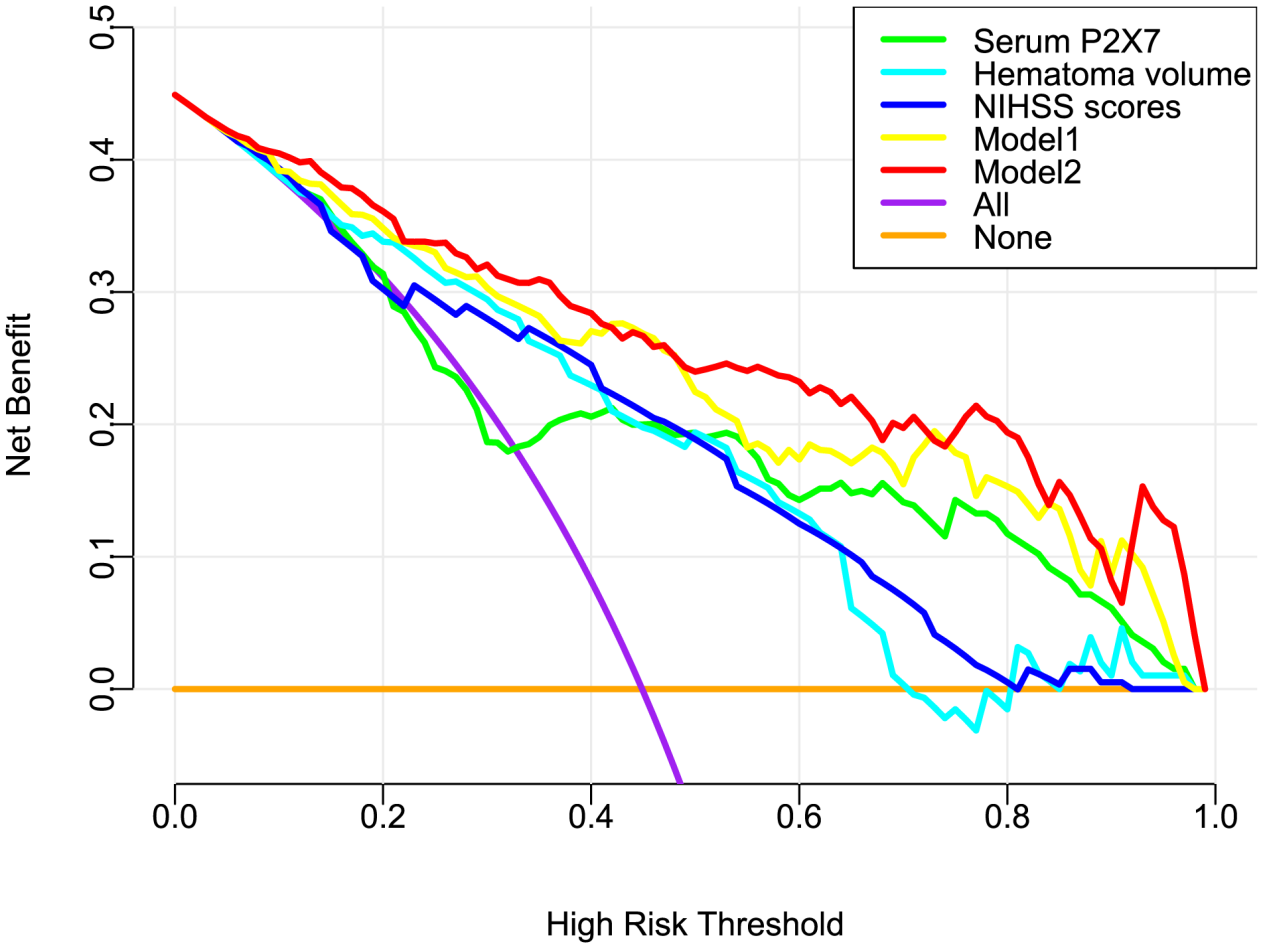
Decision curve analysis of the prognostic model in acute ICH. Model 2 (nomogram including admission serum P2X7) showed greater clinical benefit for predicting poor prognosis than model 1 (without P2X7). NIHSS, National Institutes of Health Stroke Scale.

**Figure 9:**
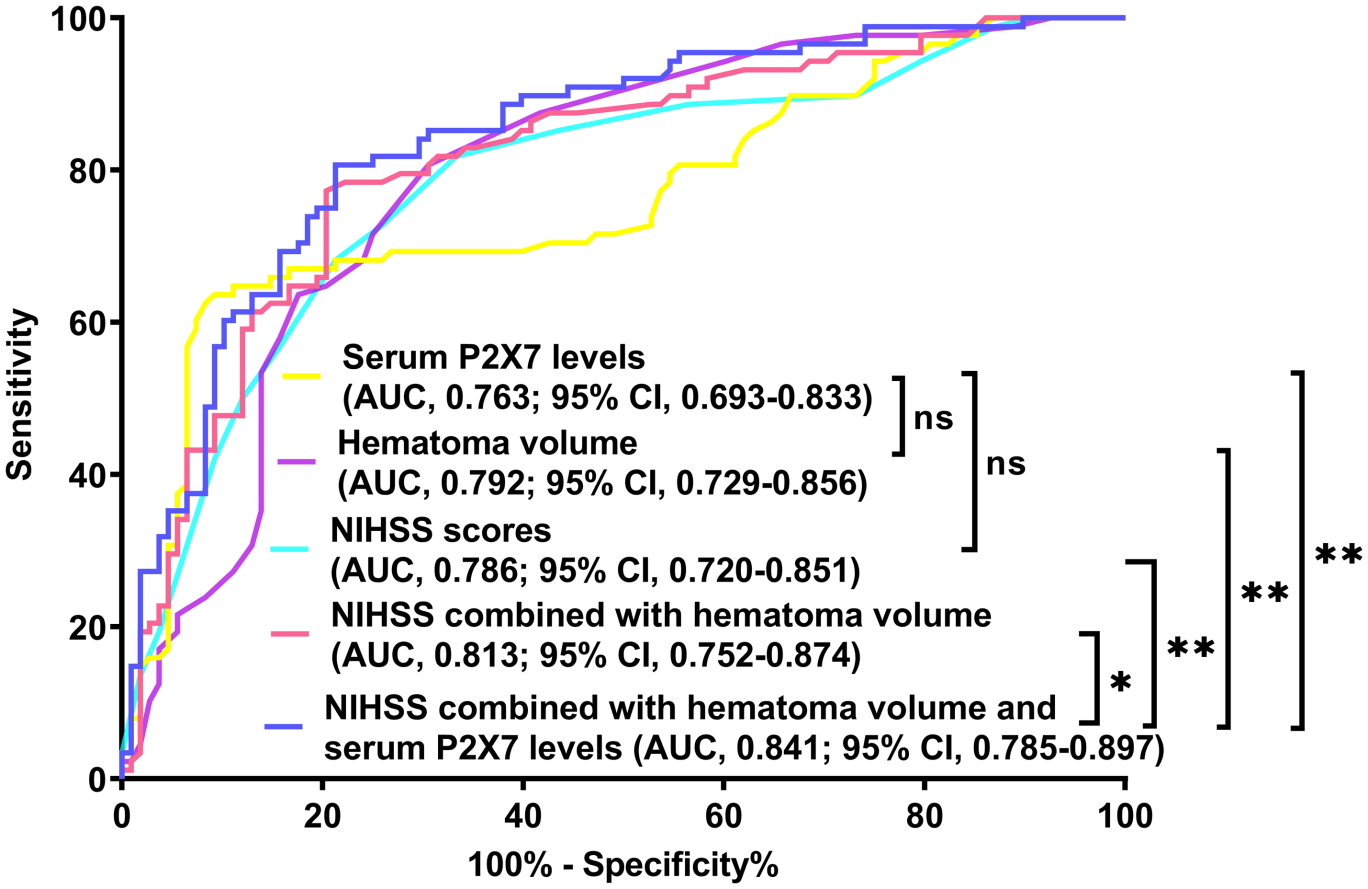
Receiver operating characteristic (ROC) curve of prognostic prediction in acute ICH. Admission serum P2X7 showed similar predictive ability to NIHSS scores and hematoma volume (both *p* > 0.05). The nomogram demonstrated the highest prognostic accuracy among all five metrics (all *p* < 0.05). NIHSS, National Institutes of Health Stroke Scale; AUC, area under the ROC curve; ns, non-significant; *p < 0.05; *p < 0.001.

**Figure 10:**
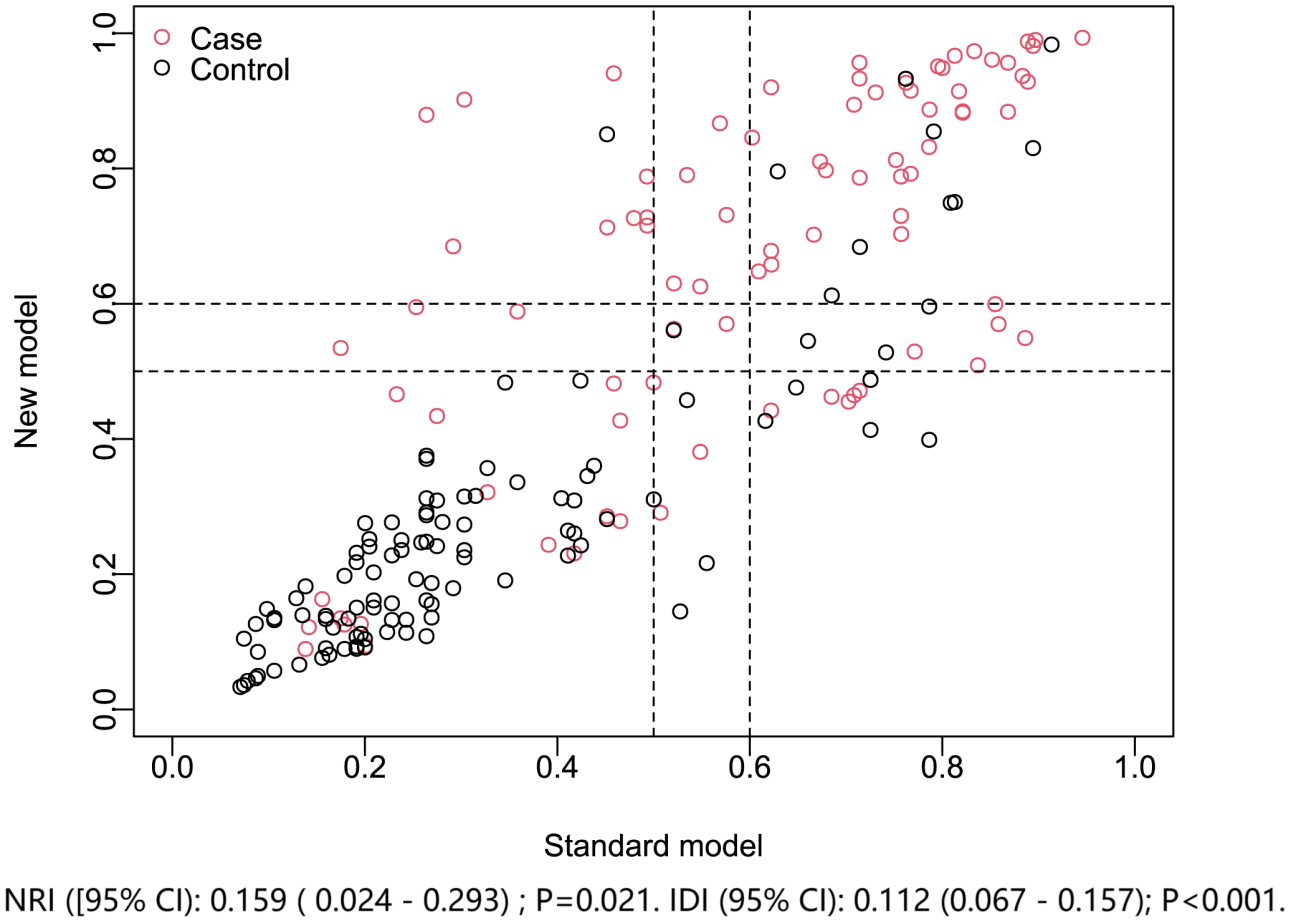
Model improvement in discriminating poor prognosis after acute ICH. The new nomogram model (including admission serum P2X7) was compared with the standard model (NIHSS plus hematoma volume). Net reclassification and integrated discrimination indices showed the nomogram provided superior prognostic performance. NRI, net reclassification improvement; IDI, integrated discrimination improvement.

In a sensitivity analysis evaluating the association between admission serum P2X7 levels and poor prognosis among all ICH patients, the E-value was 6.81 (Figure 4). In subgroup analysis, this association was not significantly moderated by other common parameters, including age, gender, and cigarette smoking (*p* > 0.05 for all interactions; Figure 5). A combined model incorporating the three predictive factors of poor prognosis (NIHSS, hematoma size, and serum P2X7) was visualized in a nomogram (Figure 6). Based on calibration curve analysis, the model predicted poor prognosis with satisfactory goodness of fit (Figure 7). As shown in Figure 8, the model integrating the three predictors, as compared to the model containing only NIHSS score and hematoma volume, had better clinical validity. In ROC curve analysis, the predictive ability of serum P2X7 levels was comparable to that of NIHSS scores and hematoma volume (both *p* > 0.05; Figure 9). In turn, the model incorporating all three predictors had markedly higher prognostic ability than models based on NIHSS score alone, hematoma volume alone, serum P2X7 alone, or the combination of NIHSS score and hematoma volume (all *p* < 0.05; Figure 9). Based on model improvement metrics, the model combining the three predictors had substantially better performance than the model based on NIHSS score and hematoma volume (Figure 10).

### 3.4 Mediation effect of serum P2X7 levels on the relationship between clinical severity and clinical outcome

Mediation analysis revealed that serum P2X7 levels partially mediated the association between NIHSS scores and poor prognosis, with a mediation effect of 24.6% (Supplementary Figure 10A). The corresponding sensitivity parameter was 0.3 (Supplementary Figure 11A). Similarly, serum P2X7 levels mediated the association between hematoma volume and poor prognosis, with a mediation effect of 26.7% (Supplementary Figure 10B) and a sensitivity parameter of 0.3 (Supplementary Figure 11B).

## 4 Discussion

To our knowledge, this is the first study to assess serum P2X7 levels in patients with ICH. The main findings are as follows: (1) serum P2X7 levels were markedly elevated at admission and remained significantly higher than those of controls up to day 10 after ICH; (2) serum P2X7 levels were strongly associated with NIHSS scores and hematoma volume, even after adjustment for potential confounding factors; (3) serum P2X7 levels were independently associated with neurological status, as reflected by mRS scores at 6 months post-ICH; (4) using a series of statistical approaches, the association between serum P2X7 levels and poor prognosis proved to be robust; (5) a prognosis prediction model encompassing NIHSS score, hematoma volume, and serum P2X7 levels performed well based on numerous statistical methods; and (6) serum P2X7 levels partially mediated the relationship between NIHSS score and hematoma volume with poor prognosis following ICH. These findings suggest that serum P2X7 may serve as a promising prognostic indicator in the clinical management of ICH.

ATP is released into the extracellular space in large amounts under pathological conditions and swiftly accumulates at focal sites in response to tissue injury and inflammation (55, 56). P2X7 receptors mediate effects of extracellular ATP, playing a critical role in transduction cascades involved in inflammasome activation, cytokine and chemokine release, T lymphocyte survival and differentiation, and cell death (57, 58). In the central nervous system, P2X7 expression is consistently upregulated in both glial cells and neuronal cells following acute brain injury. Accumulating evidence strongly indicates that P2X7 activation is a detrimental factor that contributes extensively to neuroinflammation and subsequent pathology in acute brain injury conditions (26–42).

In this study, serum P2X7 levels in ICH patients were markedly elevated upon admission, increased further at day 1, reached a peak at day 3, remained stable at day 5, and then gradually declined through post-ICH day 10, while consistently exceeding those in controls across all six time points. The blood–brain barrier is inevitably disrupted after ICH, leading to the leakage of brain-derived proteins into the peripheral circulation (59). It is therefore inferred that, following ICH, circulating P2X7 originates from brain tissues. Nevertheless, peripheral blood cells demonstrably express P2X7 protein (60, 61), which may contribute to the activation of systemic inflammatory response syndrome following ICH (62–64). This therefore suggests that ICH may stimulate peripheral blood cells to secrete P2X7, indicating that circulating P2X7 could be partially derived from these cells; however, measuring P2X7 protein expression in peripheral blood cells will be necessary to clarify its cellular origin.

Severity assessment is a crucial step in the management of all diseases. In ICH, the NIHSS score and hematoma volume are commonly used for severity appraisal and prognosis analysis (7–10). In the current study, serum P2X7 levels closely correlated with NIHSS scores and hematoma volume even after correcting for other confounding factors. Moreover, to ensure appropriate application of a linear model, RCS analysis was performed in advance to verify the assumption of linearity. Hence, our study provides preliminary evidence that serum P2X7 levels are closely associated with ICH severity.

We observed a tight correlation between serum P2X7 levels and bleeding severity, with values peaking at day 3 post-ICH and plateauing at day 5. This phenomenon is in line with temporal trends of brain edema following ICH (65). Mediation analysis provides a valuable framework to examine the underlying mechanisms that connect two variables (54). From a statistical perspective, serum P2X7 levels partially mediated the association between poor prognosis and ICH severity, as reflected by NIHSS scores and hematoma volume in this patient cohort. Taking into account that activation of P2X7 can drive neuroinflammation (26–28), and that P2X7 is now an established therapeutic target in brain diseases (43–47), it is deduced from a clinical viewpoint that P2X7 may play an inflammatory role and be implicated in the development and progression of ICH. Nevertheless, although experimental studies have explored the mechanisms of P2X7 in acute brain injury (26–42), this assumption warrants verification in future research.

Neurological deficits in patients with ICH are frequently assessed using the mRS at 6 months post-ICH (12, 13). By adopting multivariable regression analysis in conjunction with auxiliary statistical approaches—including E-value calculation for sensitivity analysis (49), RCS analysis for linearity appraisal (50), subgroup analysis for interaction assessment (51), and VIF computation for multicollinearity evaluation (52)—the independent association of serum P2X7 levels with neurological impairment, mirrored by mRS, was affirmed in this cohort of ICH patients. Serum P2X7 levels, together with two conventional determinants of poor prognosis in ICH, namely NIHSS scores and hematoma volume (7–10), were confirmed to be independently associated with 6-month poor outcomes. Moreover, serum P2X7 levels demonstrated prognostic predictive capability under the ROC curve comparable to that of NIHSS scores and hematoma volume. Importantly, two prognostic prediction systems were modeled: one based solely on NIHSS scores and hematoma volume, and another incorporating serum P2X7 in addition to these conventional predictors. By estimating the model improvement rate (53) and comparing ROC curves, the model incorporating serum P2X7 levels showed superior performance. The additive effect on established clinical and radiological predictors highlights serum P2X7 as a potential prognostic biomarker, and a model combining P2X7, NIHSS, and hematoma volume may serve as an adjunct in prognostic prediction for ICH management.

In this study, several statistical approaches supported P2X7 as a promising serological marker for severity assessment and prognosis prediction in ICH. For the ROC curve of P2X7 alone, a bimodal trend may be observed. This likely reflects the non-normal distribution of serum P2X7 levels in both the poor- and good-prognosis groups, which can give rise to such a pattern. Given that RCS analysis had already verified linearity, application of linear models such as ROC analysis was rational. Still, this bimodal trend may reduce the predictive efficiency of P2X7 alone. Therefore, a combined model integrating NIHSS scores, hematoma volume, and serum P2X7 levels was constructed, which demonstrated superior predictive ability compared with each single indicator or the NIHSS− hematoma volume combination. Moreover, the combined model showed no obvious bimodal trend. Overall, incorporating serum P2X7 helped eliminate this pattern and enhanced predictive efficiency. Alternatively, the Youden method was adopted to determine the cutoff value of serum P2X7, therefore improving predictive ability.

Two main limitations of this study warrant consideration. First, although the sample size was statistically sufficient for this cohort study, larger multicenter studies are necessary to validate the conclusions before clinical generalization. Second, although mediation effects of serum P2X7 levels were observed between poor prognosis and ICH severity, as indicated by NIHSS scores and hematoma volume, the underlying interplay requires further investigation.

## 5 Conclusion

Serum P2X7 levels are significantly elevated from admission to day 10 after ICH. They are independently associated with ICH severity and 6-month neurological status, have good prognostic predictive ability, and partially explain the connection between poor prognosis and ICH severity. Accordingly, serum P2X7 may represent a promising biomarker for severity assessment and outcome prediction in ICH.

## Data Availability

The raw data supporting the conclusions of this article will be made available by the authors, without undue reservation.

## Footnotes

1
www.r-project.org
