# Supplementary material for: Serum P2X7 as a prognostic biomarker in acute supratentorial intracerebral hemorrhage: a two-center observational study
Source: Front Neurol. 2026 Jan 14;16:1696189. doi: 10.3389/fneur.2025.1696189 (PMC12847250; doi:10.3389/fneur.2025.1696189)
Supplement: Supplementary file 1 [file Supplementary_file_1.docx]

**Figure legends**

**Supplementary Figure 1**

Bland-Altman plot delineating concordance of two measurements.

Dual measurements of serum P2X7 levels had a satisfactory concordance in the total data (intraclass correlation coefficient=0.993).

SD means standard deviation.

**Supplementary Figure 2**

Bivariate correlation analysis of various markers in acute intracerebral hemorrhage.

National Institutes of Health Stroke Scale scores, hematoma volume and modified Rankin Scale scores were firmly correlated with serum P2X7 levels at admission and at days 1, 3, 5, 7 and 10 in patients with acute intracerebral hemorrhage (all P<0.001).

NIHSS indicates National Institutes of Health Stroke Scale; mRS, modified Rankin Scale.

**Supplementary Figure 3**

Linearity relationship between admission serum P2X7 levels and other variables in acute intracerebral hemorrhage.

Under restricted cubic spline, admission serum P2X7 levels were linearly related to National Institutes of Health Stroke Scale scores (P for nonlinear >0.05; A), hematoma volume (P for nonlinear >0.05; B), and modified Rankin Scale scores (P for nonlinear >0.05; C) in all patients with acute intracerebral hemorrhage.

NIHSS signifies National Institutes of Health Stroke Scale; mRS, modified Rankin Scale.

**Supplementary Figure 4**

Serum P2X7 levels at admission and other variables in acute intracerebral hemorrhage.

Serum P2X7 levels at admission were significantly positively related to National Institutes of Health Stroke Scale scores (P<0.001; A), hematoma volume (P<0.001; B) and modified Rankin Scale scores (P<0.001; C) among all patients with acute intracerebral hemorrhage.

NIHSS denotes National Institutes of Health Stroke Scale; mRS, modified Rankin Scale.

**Supplementary Figure 5**

Serum P2X7 levels at different time points among seven subgroups with different modified Rankin Scale scores at six months after acute intracerebral hemorrhage.

Modified Rankin Scale ranged from 0 to 6 and patients for serial samplings were divided into seven subgroups according to the scores from 0 to 6. Serum P2X7 levels at six time points were obviously different among seven subgroups with different modified Rankin Scale scores at six-month mark following acute intracerebral hemorrhage in patients for continuous samplings, with the higher the scores and the higher the levels (all ^**^P<0.001).

mRS stands for modified Rankin Scale.

**Supplementary Figure 6**

Admission serum P2X7 levels among patients with different six-month modified Rankin Scale scores subsequent to acute intracerebral hemorrhage.

Modified Rankin Scale spanned from 0 to 6 and patients for continuous samplings were assigned into seven subgroups in accordance with the scores from 0 to 6. Serum P2X7 levels at admission were substantially increased in order of modified Rankin Scale scores from 0 to 6 in all patients with acute intracerebral hemorrhage (P overall <0.001).

mRS means modified Rankin Scale; ns, non-significant. ^***^P<0.001; ^**^P<0.01; ^*^P<0.05.

**Supplementary Figure 7**

Serum P2X7 levels at different time points between patients with poor prognosis and those without the event following acute intracerebral hemorrhage.

Serum P2X7 levels at six time points were markedly higher in cases with poor prognosis than in the remainders among acute intracerebral hemorrhage patients for continuous sampling (all ^**^P<0.01).

**Supplementary Figure 8**

Prediction ability of serum P2X7 levels at different time points on poor prognosis of patients with acute intracerebral hemorrhage.

Area under the receiver operating characteristic curve of serum P2X7 levels at day 0 (admission) resembled those of serum P2X7 levels at the other days following acute intracerebral hemorrhage among patients for dynamic measurements (all P>0.05).

AUC means area under curve; 95% CI, 95% confidence interval; ns, nonsignificant.

**Supplementary Figure 9**

Admission serum P2X7 levels and poor prognosis in all patients after acute intracerebral hemorrhage.

Among all patients with acute intracerebral hemorrhage, cases with the development of poor prognosis, as opposed to those without the event, displayed pronouncedly heightened serum P2X7 levels at admission (P<0.001; A); under the receiver operating characteristic curve, admission serum P2X7 levels were satisfactorily predictive of poor prognosis and the optimal threshold value was selected by employing the Youden method (B).

AUC indicates area under curve; 95% CI, 95% confidence interval. Red arrow signifies cutoff value.

**Supplementary Figure 10**

Mediation effects of serum P2X7 levels on relevance of severity parameters with neurological outcome in acute intracerebral hemorrhage.

Mediation analysis showed that serum P2X7 levels in part mediated connections between National Institutes of Health Stroke Scale (A), along with hematoma volume (B), and neurological outcome of all patients with acute intracerebral hemorrhage.

NIHSS denotes National Institutes of Health Stroke Scale; Volume, hematoma volume.

**Supplementary Figure 11**

Sensitivity analysis regarding mediation effect of serum P2X7 levels on association of severity indexes with neurological outcome following acute intracerebral hemorrhage.

Serum P2X7 levels effectively mediated correlations of National Institutes of Health Stroke Scale (A) and hematoma volume (B) with neurological outcome in all patients with acute intracerebral hemorrhage.


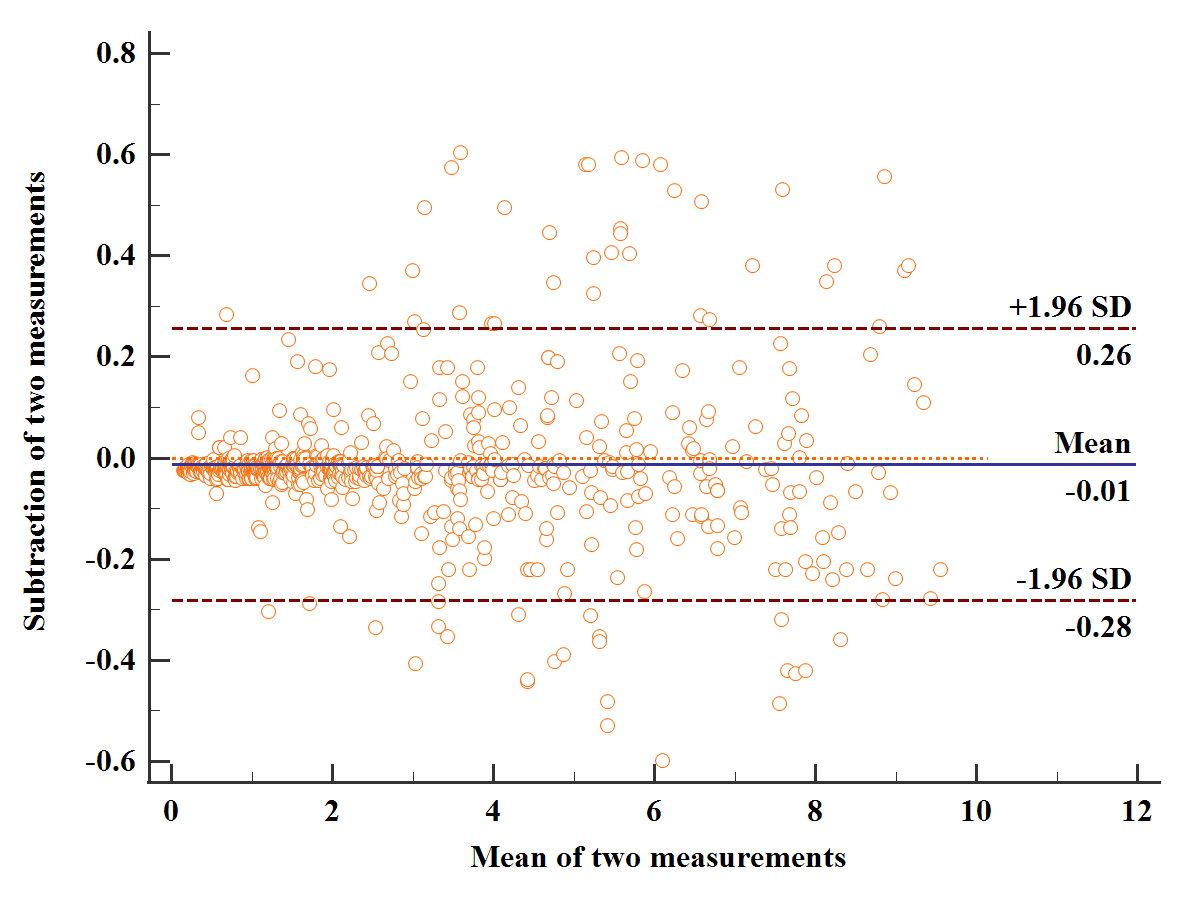


**Supplementary Figure 1**


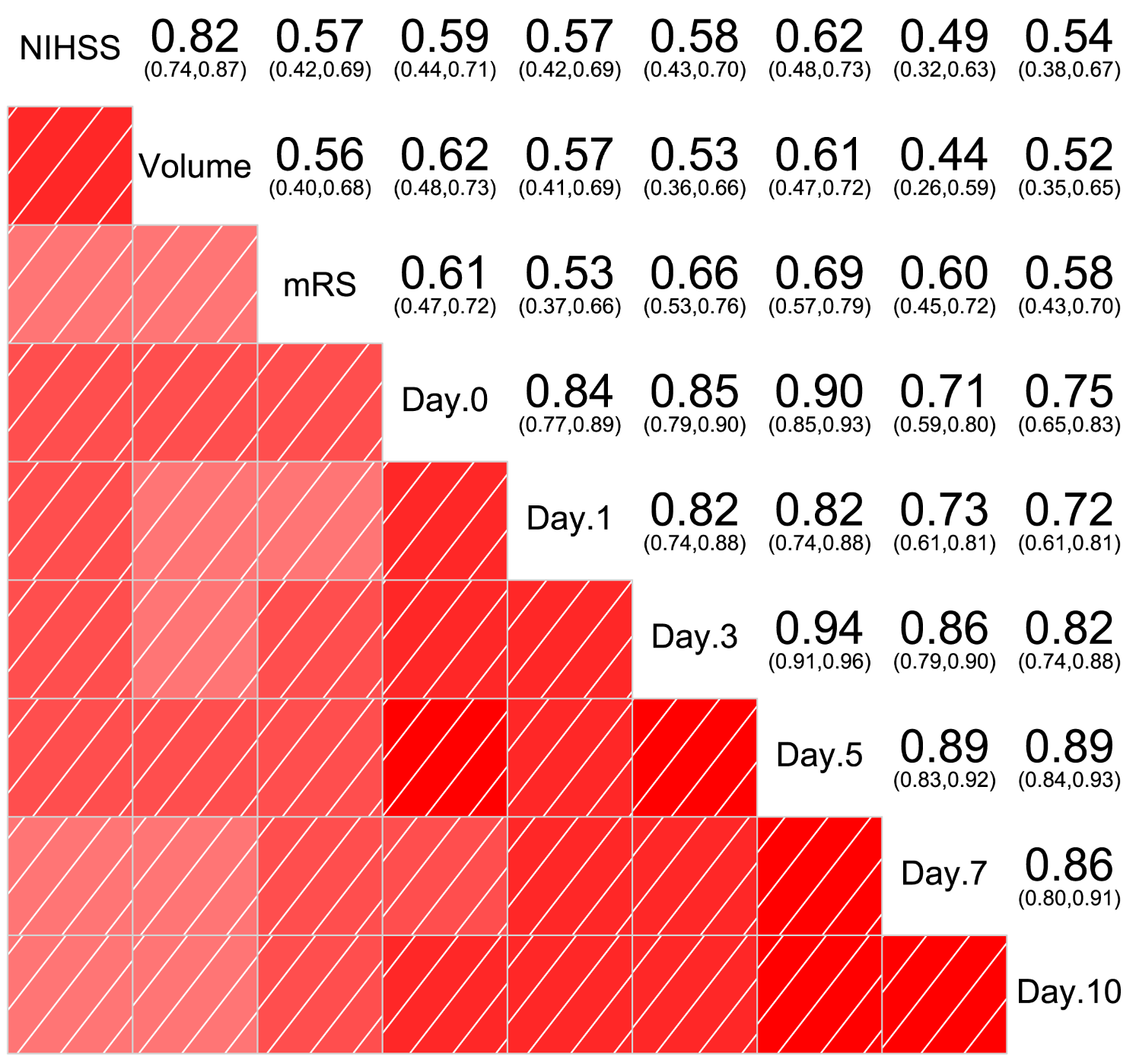


**Supplementary Figure 2**


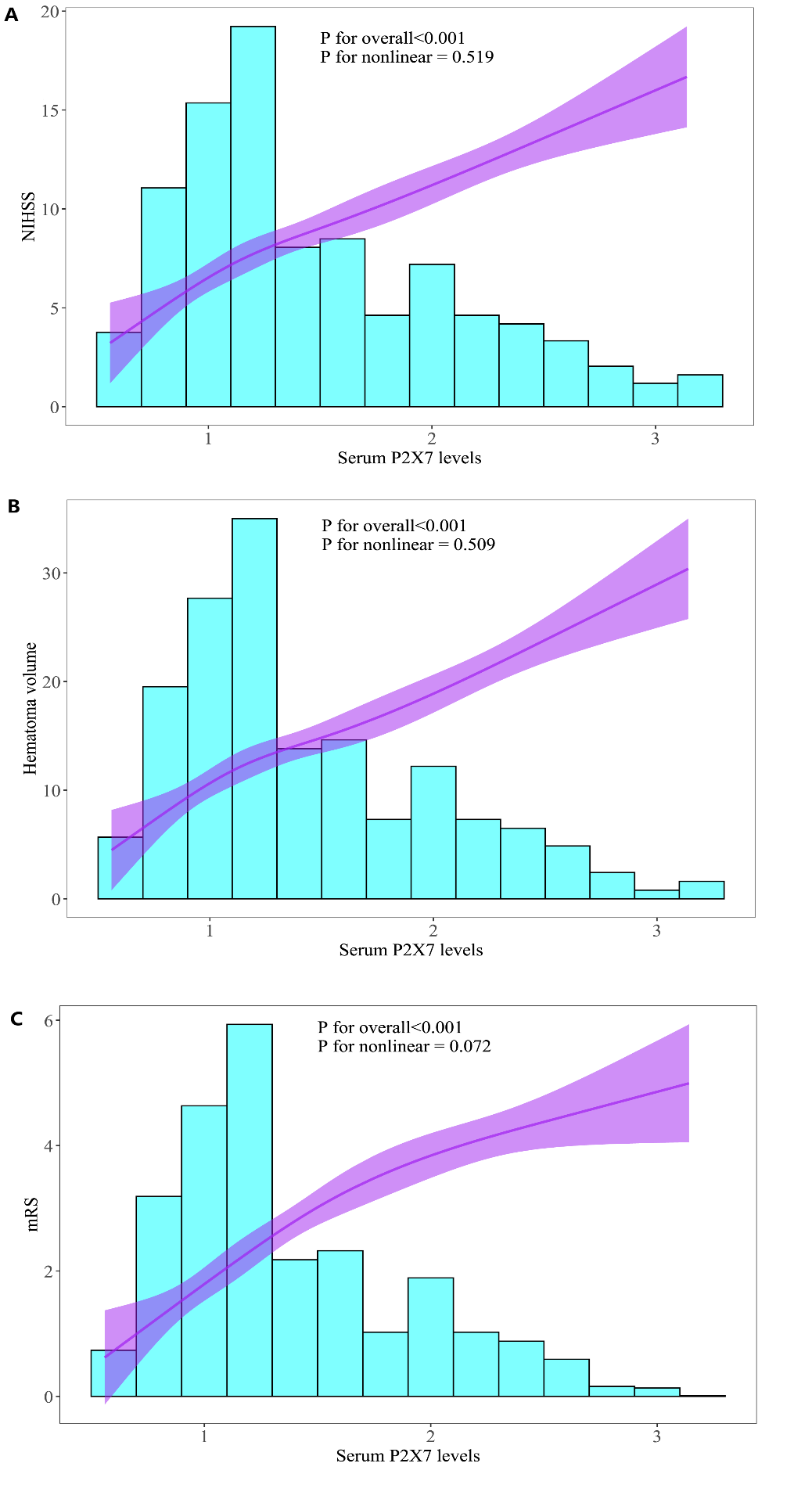


**Supplementary Figure 3**


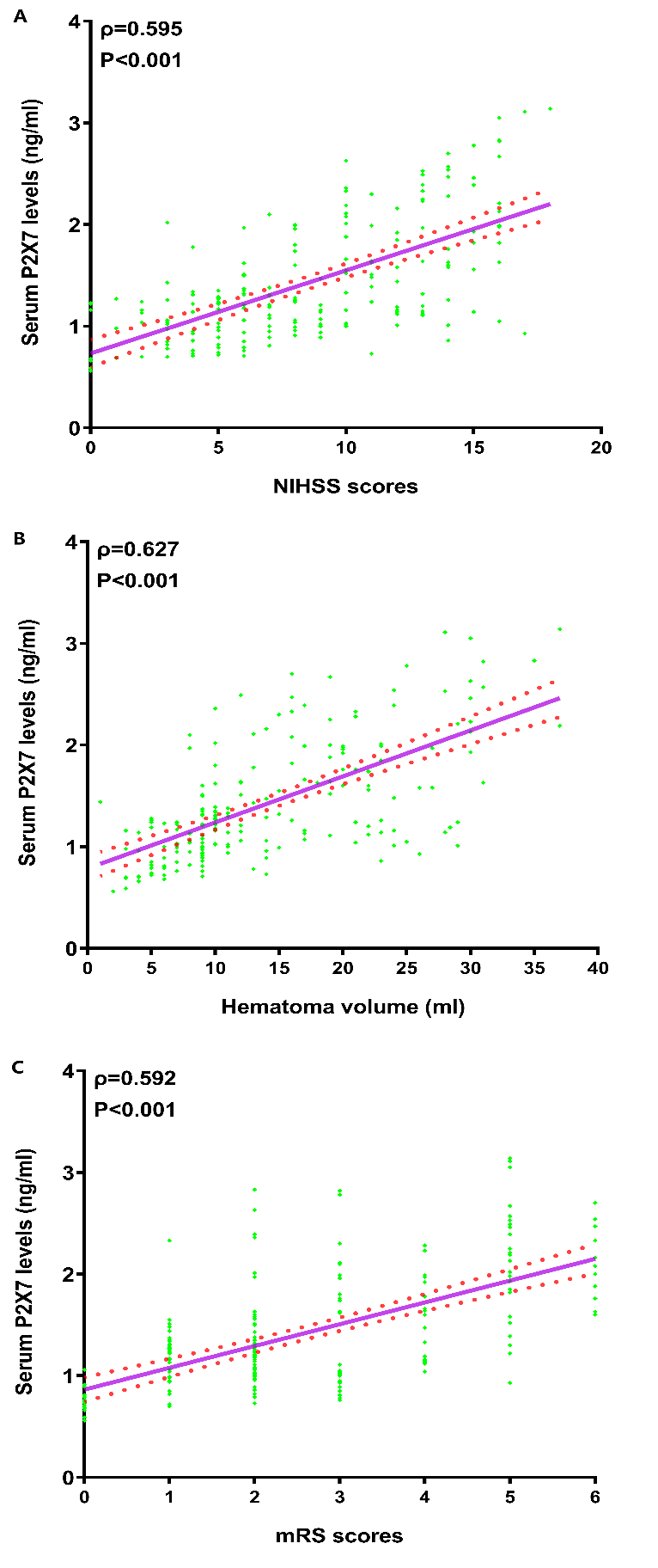


**Supplementary Figure 4**


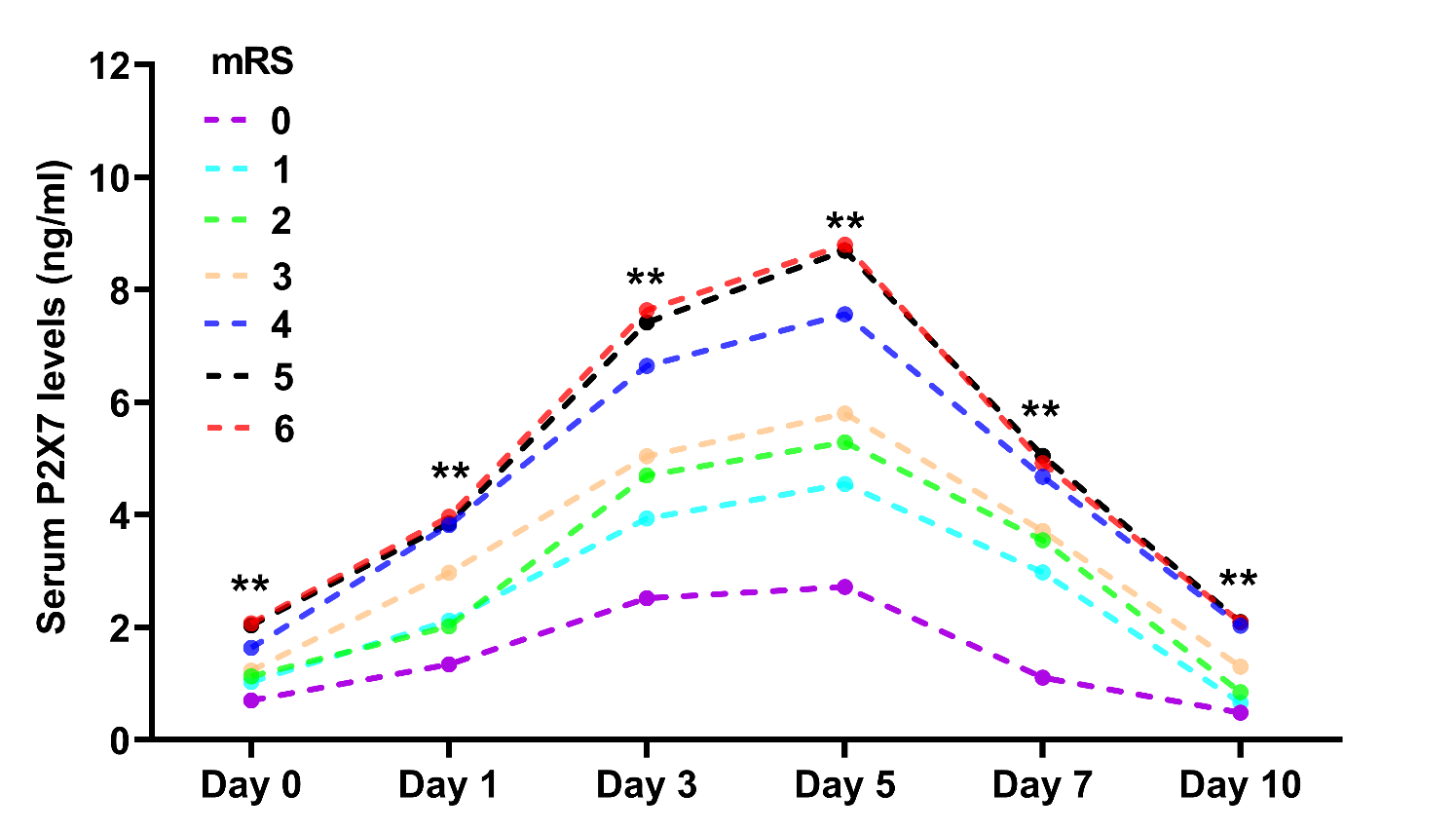


**Supplementary Figure 5**


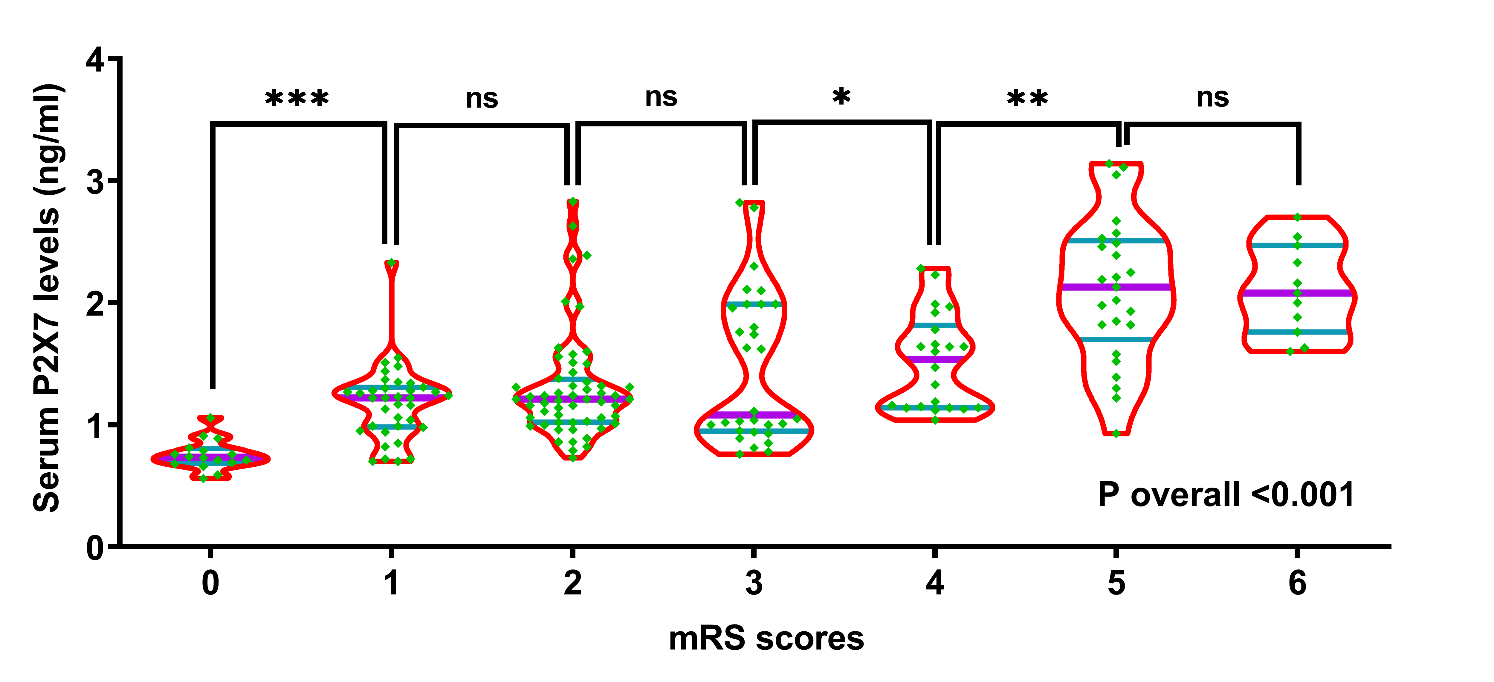


**Supplementary Figure 6**


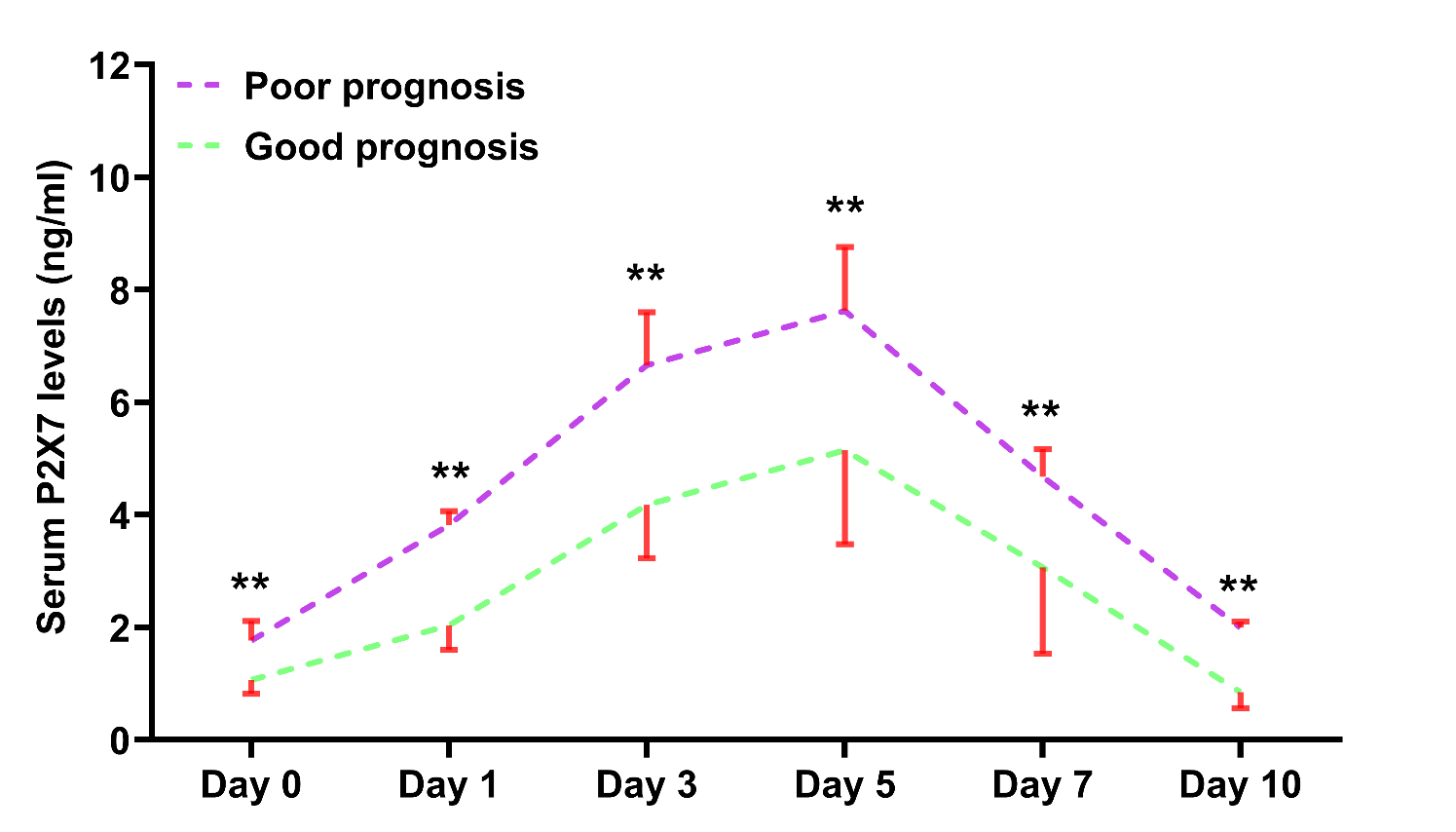


**Supplementary Figure 7**


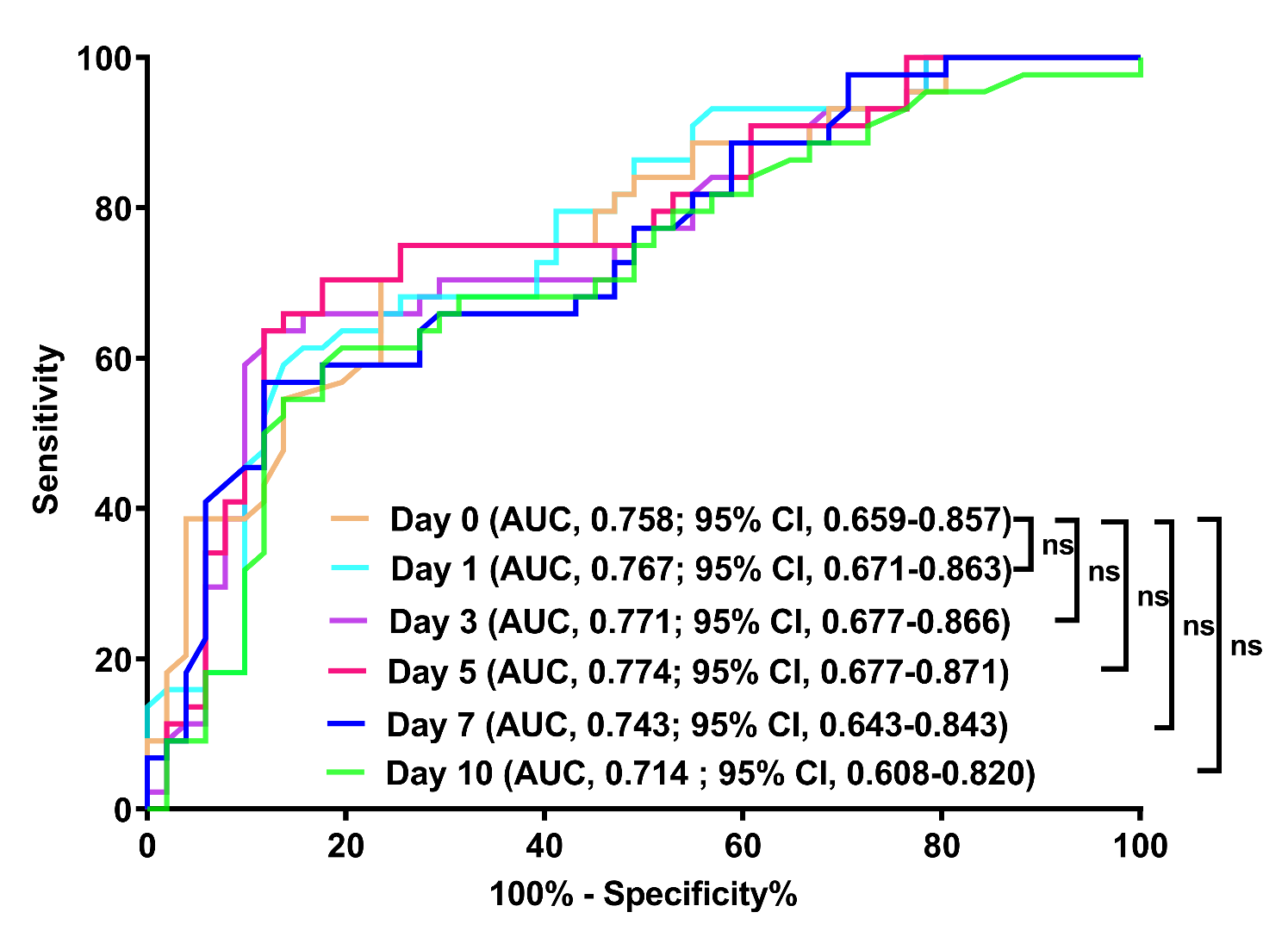


**Supplementary Figure 8**


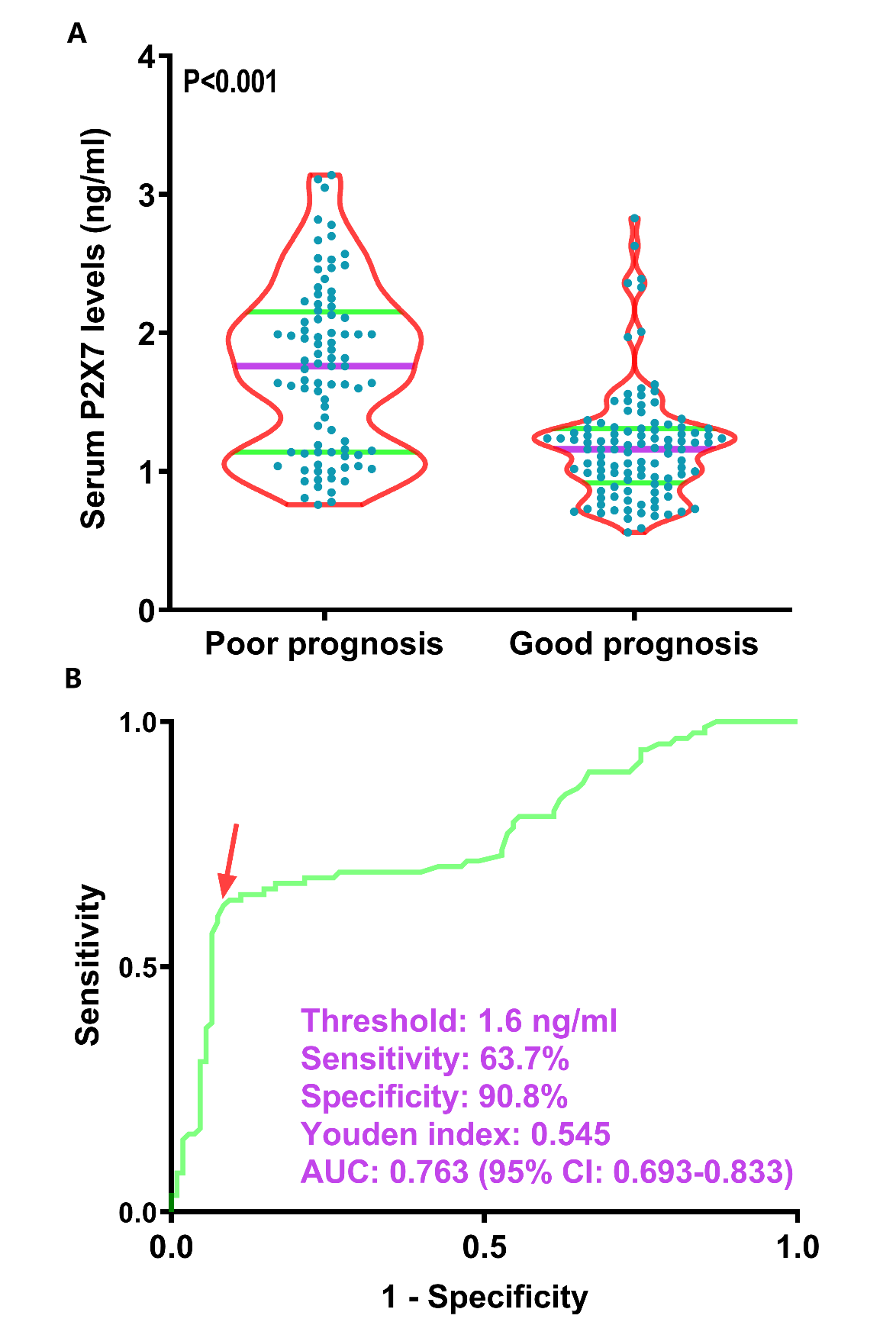


**Supplementary Figure 9**


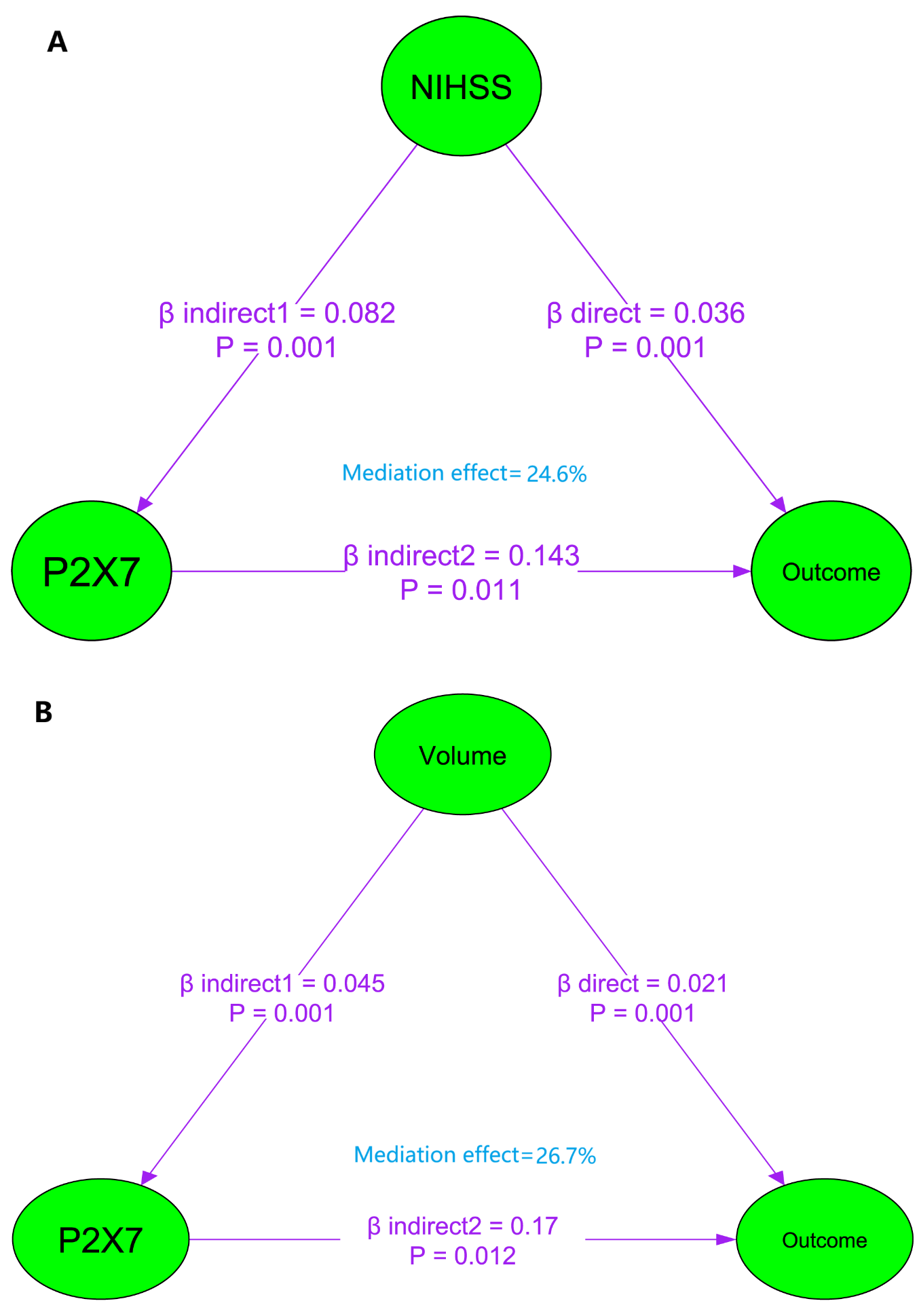
 **Supplementary Figure 10**


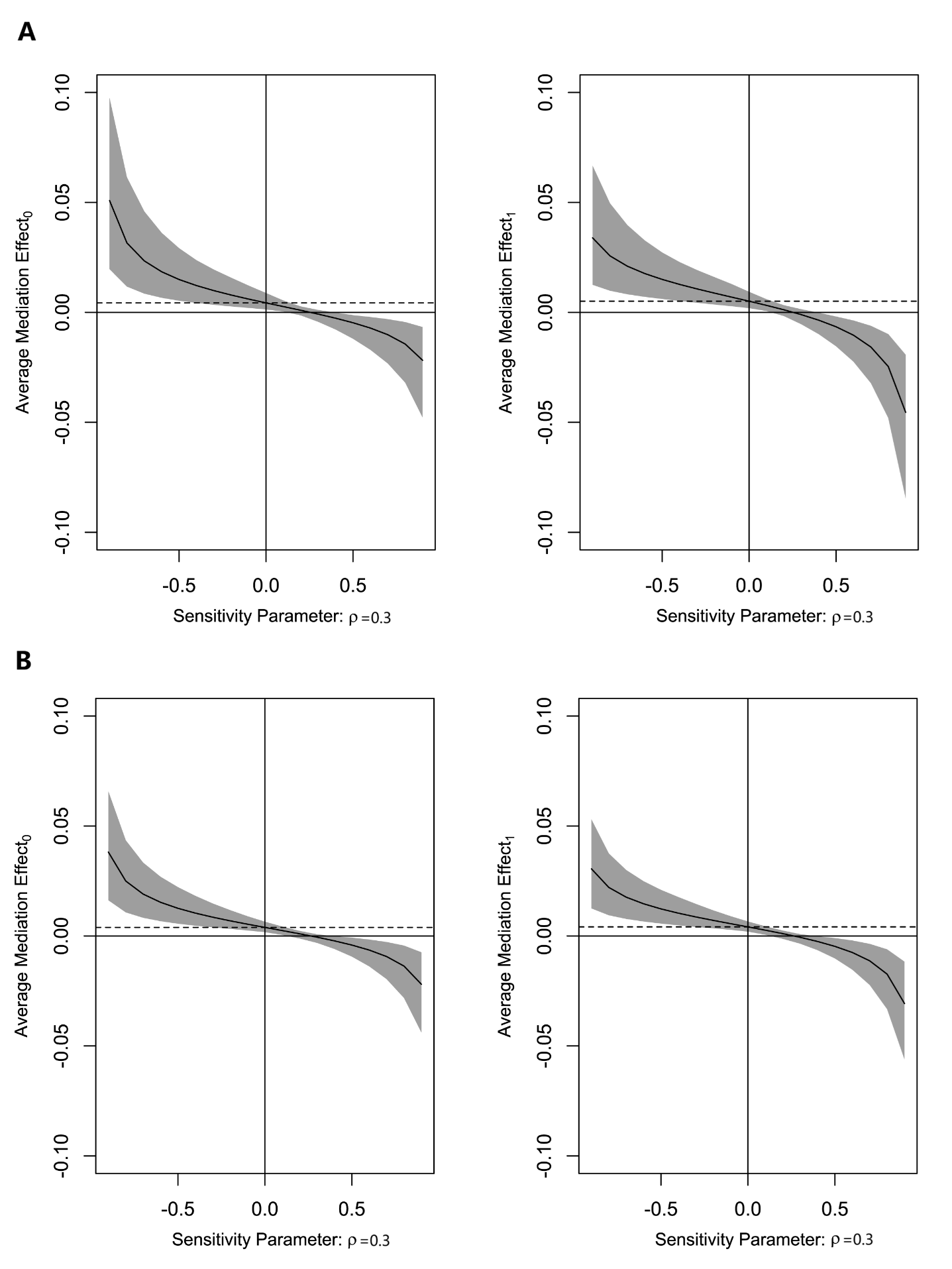


**Supplementary Figure 11**
